# Inequalities in all-cause and cause-specific mortality across the life course by wealth and income in Sweden: a register-based cohort study

**DOI:** 10.1093/ije/dyaa053

**Published:** 2020-05-07

**Authors:** S Vittal Katikireddi, Claire L Niedzwiedz, Ruth Dundas, Naoki Kondo, Alastair H Leyland, Mikael Rostila

**Affiliations:** d1 MRC/CSO Social & Public Health Sciences Unit, University of Glasgow, UK; d2 Institute of Health & Wellbeing, Glasgow, UK; d3 Graduate School of Medicine, University of Tokyo, Japan; d4 Department of Public Health Sciences, Stockholm University, Sweden; d5 Centre for Health Equity Studies, Stockholm University/Karolinska Institutet, Sweden

**Keywords:** Social determinants of health, mortality, health equity, socio-economic factors, population register

## Abstract

**Background:**

Wealth inequalities are increasing in many countries, but their relationship to health is little studied. We investigated the association between individual wealth and mortality across the adult life course in Sweden.

**Methods:**

We studied the Swedish adult population using national registers. The amount of wealth tax paid in 1990 was the main exposure of interest and the cohort was followed up for 18 years. Relative indices of inequality (RII) summarize health inequalities across a population and were calculated for all-cause and cause-specific mortality for six different age groups, stratified by sex, using Poisson regression. Mortality inequalities by wealth were contrasted with those assessed by individual and household income. Attenuation by four other measures of socio-economic position and other covariates was investigated.

**Results:**

Large inequalities in mortality by wealth were observed and their association with mortality remained more stable across the adult life course than inequalities by income-based measures. Men experienced greater inequalities across all ages (e.g. the RII for wealth was 2.58 [95% confidence interval (CI) 2.54–2.63) in men aged 55–64 years compared with 2.29 (95% CI 2.24–2.34) for women aged 55–64 years), except among the over 85s. Adjustment for covariates, including four other measures of socio-economic position, led to only modest reductions in the association between wealth and mortality.

**Conclusions:**

Wealth is strongly associated with mortality throughout the adult life course, including early adulthood. Income redistribution may be insufficient to narrow health inequalities—addressing the increasingly unequal distribution of wealth in high-income countries should be considered.

**Key Messages**

- Wealth inequalities are rising in many countries internationally, but studies of the relationship between wealth and mortality have been limited to older people and the health implications remain uncertain.
- In the largest study to date, wealth was associated with all-cause mortality in all adult age groups, among both men and women.
- Adjustment for four other measures of socio-economic position (education, individual income, household income and social class), as well as other covariates, resulted in only modest attenuation.
- Progressive income taxation may be inadequate to address health inequalities, with consideration for measures that narrow wealth inequalities needed as well.

## Background

There is growing political and public concern about increasing wealth inequality, with eight people now owning the same wealth as half of the world’s population.^1^^,^^2^ High-profile academic debates have considered the potential implications of the growing concentration of wealth on economic outcomes.^3^ The social circumstances in which people live, from early life to old age, have large health impacts.^4^ However, there has been limited investigation of what the implications of increasing wealth inequality might be for public health.^5^ The limited research has predominantly focused on older adults.^6–8^

The social determinants of health underpin health inequalities—the repeated finding that socially disadvantaged groups tend to experience worse health in a way that is both unjust and avoidable.^9^^,^^10^ Health inequalities research has measured socio-economic position using a range of approaches.^11–14^ While education level and social class are widely used, these variables are indirectly related to an individual’s material resources. Furthermore, they may be less relevant for specific age groups—e.g. education level typically remains unchanged after early adulthood. Income is a more specific measure of the capacity of an individual to buy resources which directly or indirectly yield health benefits. Wealth is clearly related to income, but includes holding a stock of assets which may not be consumed during their use (e.g. ownership of housing, land and shares).^5^ It is possible that the importance of income and wealth for health outcomes differs, and these relationships may vary across the life course. For example, income inequalities among older people may decrease following retirement while wealth inequalities remain substantial. For this reason, it is often suggested that wealth may be particularly important in old age but not among young adults.^11^

We investigate the association between individual wealth and mortality across the adult life course in the Swedish population. Furthermore, we compare the magnitude of these inequalities with those measured by individual and household income and report inequalities across different causes of death.

## Methods

### Population

Data were collated from the population register, the death register and the 1990 Swedish census. Since the last census in 1990, the population register has been used to provide a complete enumeration of the resident population of Sweden.^15^ These data were deterministically linked (on the basis of the unique population identifier) to the Longitudinal Database for Health Insurance and Labour Market Studies (LISA) which provides information on both individual and household measures of income.^16^ Mortality follow-up data were available for the linked datasets up to the end of 2007, with individuals censored for migration and death. All datasets include the unique personal identifier as a compulsory field, therefore the potential for missed linkages between the same individual is believed to be negligible.^15^^,^^17^

We study all adults (aged ≥25 years) in the Swedish population from 1990 onwards, classified into six groups, based on baseline age: 25–39, 40–54, 55–64, 65–74, 75–84 and 85+ years. The primary analyses excluded <0.01% of the population (246 individuals) due to either missing income or wealth data. Highest educational attainment was not available for those aged 85+ years and therefore this group was excluded from analyses adjusting for other socio-economic variables.

### Exposures

Historically, the Swedish government administered a tax on individual wealth.^18^^,^^19^ The taxation rate was low, calculated based on the amount of wealth an individual owned above a minimum threshold. In 1990, the minimum threshold for payment was 800 000 SEK (equivalent to ∼£68 542 or US $87 398 in November 2018) and the minimum taxation rate was 1.5% (rising to a 3.0% maximum).^19^ Taxable assets were calculated as the sum of individual assets minus any outstanding liabilities at the end of the fiscal year. The assets included real estate (based on value assessed in the previous year, with outstanding mortgages accounted for), high-value personal items (such as cars, jewellery and boats), bank accounts, shares, government bonds and property annuities.

Wealth was therefore measured by the amount of wealth tax paid by an individual in 1990. At that time, 34.9% of the study population had wealth assets below the threshold. For this reason, and to account for differences in the distribution across age–sex groups, a rank-based approach to the analysis of inequalities was adopted by calculating the relative index of inequality (RII) and the slope index of inequality (SII).^20^ These involve ranking everyone in each age–sex group from the most advantaged (i.e. paying the highest wealth tax) to the least advantaged (i.e. not paying any) and standardizing the rank from zero to one. The RII is the coefficient obtained from a Poisson regression of this standardized rank on mortality and can be interpreted as the relative risk for the hypothetically most disadvantaged person in society compared to the most advantaged, taking into account the entire distribution of data. More detail is provided in the Supplementary Material (pp 2), available as supplementary data at *IJE* online. For cause-specific mortality, the SII was calculated using the formula 2xASMRx(RII − 1)/(RII + 1), where ASMR is the age-standardized mortality rate (to the WHO European Standard population) and indicates the absolute risk difference between the hypothetically most and least advantaged person.^21^ In additional analyses, we explored the consistency of the pattern of findings to an alternative categorization of our wealth measure. For the youngest two age groups we compared all-cause mortality rates for those paying some wealth tax vs those paying none and for older groups we assessed differences between quartiles.

Two different measures of income from 1990 were analysed for comparative purposes: first, an individual’s net disposable income (total income from paid employment and benefits minus taxes) and second, household income (equivalized for household composition). These variables were categorized in two different ways: first, using a rank-based approach that allows calculation of RIIs and SIIs for the purpose of comparison to wealth; and second, as quintiles so that they could be incorporated as a covariate while allowing for non-linear relationships with mortality.

Other dimensions of socio-economic position were incorporated into the analysis as covariates, to investigate whether wealth was independently associated with mortality, in addition to other commonly used socio-economic measures. Highest educational attainment generally reflects early adulthood socio-economic position, since only a low proportion of the population gain further qualifications after this stage of the life course. Education was categorized into three groups: degree or higher, upper secondary and compulsory education only. Social class is an occupation-based measure defined by Statistics Sweden and was classified in eight categories: upper non-manual, intermediate non-manual, lower non-manual, skilled manual, unskilled manual, farmers and farm labourers, self-employed, unclassified employees and economically inactive. Lastly, we also included country of birth (Swedish born and foreign born), geographical region, number of children aged <18 years (grouped as none, one, two and three plus) and number of adult children aged ≥18 years within the household (none, one and two plus) as factors that could potentially both mediate and confound observed associations.

### Outcomes

Our primary outcome was all-cause mortality from 1 January 1990 to 31 December 2007 (the last date for which follow-up was available). Secondary analyses investigated cause-specific mortality, categorized into the following broader groupings: infection, cancer, diabetes, dementia, circulatory diseases, respiratory disease, alcohol, drugs and accidents and violence. Common causes of death were further sub-classified: circulatory diseases were separated into ischaemic heart disease (IHD), stroke and other; cancer into lung, stomach, colon, prostate, breast, female reproductive cancers and other; and accidents and violence into road traffic incidents, suicide, homicide and other. The International Classification of Disease (ICD)-9 and ICD-10 codes used are detailed in the Supplementary Material (pp 2).

### Statistical analysis

In the first phase of the analysis, we used Poisson regression with robust standard errors to calculate RIIs for wealth, individual income and household income univariately. Age at baseline (5-year bands up to 90+) and follow-up year were adjusted for, with stratification by sex, as the relationship between income and health is known to vary between males and females. Person-years at risk were used as the offset and individuals migrating outside of Sweden were censored from the analysis. In the second phase, we adjusted for covariates to investigate the extent to which wealth was independently associated with mortality inequalities. To provide adequate statistical precision, the last phase of the analysis assessed cause-specific mortality (using RIIs and SIIs) in the whole study population, stratified by sex and adjusting for baseline age. All analyses were carried out using Stata 13.1, with Microsoft Office 2010 and RStudio v1.1.456 used to create figures.

Ethical permission (No. 02–481) was provided by the Regional Ethics Committee at Karolinska Institutet in Stockholm.

## Results

The study population comprised 6.04 million people (48.5% men) and experienced 1.7 million deaths during 97.1 million person-years of follow-up (Supplementary Material, pp 4–5). Missing data were minimal for the main analyses and <10% for all adjusted analyses. The amount of wealth tax paid varied substantially by age-group and sex, with more young people not having enough wealth to pay any tax (Supplementary Material pp 6). Whereas younger men were more likely not to pay any wealth tax than younger women, the reverse was true in the oldest age groups.

**Figure 1:**
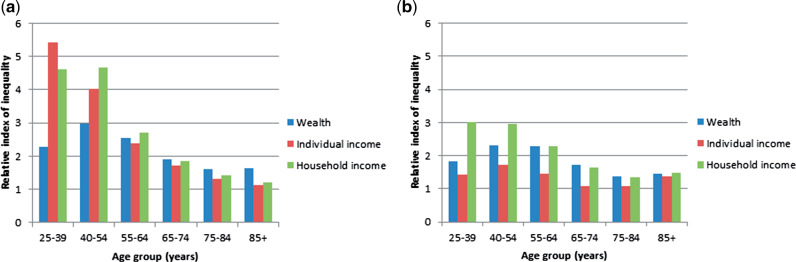
Relative indices of inequality (RII) in all-cause mortality in Swedish adults by wealth, individual income and household income in (a) men and (b) women. Separate regression models were estimated for each age–sex group and each exposure; models were adjusted for age (5-year bands) and follow-up only.

Large mortality inequalities by wealth were seen for all age and sex groups (Figure 1 and Table 1). In men, mortality-based inequalities by income were particularly large in young age groups whereas inequalities by wealth differed less by age. For example, the RII by individual income was 5.44 [95% confidence interval (CI) 5.20–5.70] in 25–39 year old men and 1.15 (95% CI 1.11–1.19) in 85+ year olds, whereas the respective figures for wealth-based mortality inequalities were 2.29 (95% CI 2.18–2.40) and 1.66 (95% CI 1.61–1.71). Relative inequalities in mortality decrease with age across all measures of socio-economic position, which is perhaps due to selective mortality^22^ and increased risk of mortality due to external causes at younger ages, especially among men. Compared with the other measures of material resources, wealth seems to become more important in older age groups, which may be due to older people having to rely more on their wealth post-retirement. In women, household income and wealth-based inequalities in mortality were larger than for individual income in younger adults. In 25–39 year old women, RIIs were 3.02 (95% CI 2.84–3.21), 1.82 (95% CI 1.72–1.93) and 1.44 (95% CI 1.36–1.52), respectively. Furthermore, household income and wealth appeared to be relatively more important for women in comparison to individual income.

**Table 1.** Relative indices of inequality (RIIs) in all-cause mortality by wealth, individual income and household income **a**djusted for age (5-year bands) and follow-up only. L95 and U95 are lower 95% and upper 95% confidence intervals respectively; *P* values <0.001 for all models

	25–39 years			40–54 years			55–64 years			65–74 years			75–84 years			85+ years		
	RII	L95	U95	RII	L95	U95	RII	L95	U95	RII	L95	U95	RII	L95	U95	RII	L95	U95
**Men**																		
Wealth	2.29	2.18	2.40	2.99	2.92	3.06	2.58	2.54	2.63	1.95	1.92	1.97	1.63	1.60	1.65	1.66	1.61	1.71
Individual income	5.44	5.20	5.70	4.04	3.94	4.14	2.41	2.37	2.46	1.75	1.73	1.77	1.35	1.33	1.37	1.15	1.11	1.19
Ratio individual income/wealth	2.38			1.35			0.93			0.90			0.83			0.69		
Household income	4.63	4.41	4.86	4.70	4.59	4.82	2.76	2.70	2.81	1.89	1.87	1.92	1.45	1.43	1.48	1.23	1.19	1.27
Ratio household income/wealth	2.03			1.57			1.07			0.97			0.89			0.74		
**Women**																		
Wealth	1.82	1.72	1.93	2.33	2.26	2.40	2.29	2.24	2.34	1.75	1.73	1.77	1.40	1.38	1.42	1.46	1.43	1.49
Individual income	1.44	1.36	1.52	1.73	1.68	1.78	1.44	1.41	1.48	1.08	1.06	1.09	1.09	1.08	1.11	1.42	1.39	1.45
Ratio individual income/wealth	0.79			0.74			0.63			0.61			0.78			0.97		
Household income	3.02	2.84	3.21	2.96	2.88	3.05	2.31	2.26	2.37	1.65	1.63	1.67	1.36	1.34	1.38	1.53	1.50	1.57
Ratio household income/wealth	1.66			1.27			1.01			0.94			0.97			1.05		

Characteristics of the study sample used for adjusted analyses are shown in the Supplementary Material (pp 7). As expected, younger people tended to have attained greater levels of education and were less likely to be economically inactive. Adjustment for each of the four other measures of socio-economic position (education, individual income, household income, occupational social class) in turn and all together resulted in only modest attenuation (Table 2 and Supplementary Material pp 8–13). In general, adjustment for other measures of socio-economic position altered the observed associations more in younger than older age groups. In working-age adults (<65 years old), adjustment for occupational social class generally led to the greatest reduction in wealth-based RIIs, in comparison to adjustment for other socio-economic measures. In contrast, adjustment for income-based measures (individual income in men and household income in women) led to slightly greater attenuation in the 65–74 years age group.

**Table 2.** Relative indices of inequality (RIIs) in all-cause mortality by wealth, adjusting for education, individual income, household income and occupational social class. Crude model adjusted for age (5-year bands) and follow-up, other models adjusted (Adj.) for four different measures of socio-economic position (SEP) in turn and together; L95 and U95 are lower 95% and upper 95% confidence intervals respectively; *P* values <0.001 for all models

	25–39 years			40–54 years			55–64 years			65–74 years		
	RII	L 95	U 95	RII	L 95	U 95	RII	L 95	U 95	RII	L 95	U 95
**Men**												
Crude	2.32	2.20	2.44	2.95	2.88	3.02	2.50	2.45	2.55	1.82	1.80	1.84
Adj. for individual income	2.17	2.06	2.29	2.58	2.51	2.64	2.24	2.20	2.28	1.64	1.62	1.67
Adj. for household income	2.14	2.03	2.25	2.42	2.36	2.48	2.16	2.11	2.20	1.64	1.62	1.66
Adj. for education	2.16	2.05	2.27	2.84	2.77	2.91	2.42	2.37	2.46	1.76	1.74	1.79
Adj. for social class	1.90	1.81	2.01	2.35	2.29	2.41	2.14	2.10	2.18	1.76	1.73	1.78
Adj. for all SEP measures	1.78	1.69	1.88	2.07	2.02	2.12	1.93	1.89	1.96	1.56	1.54	1.59
**Women**												
Crude	1.84	1.73	1.95	2.31	2.24	2.38	2.24	2.19	2.29	1.69	1.67	1.71
Adj. for individual income	1.90	1.79	2.03	2.29	2.22	2.36	2.15	2.10	2.20	1.66	1.63	1.68
Adj. for household income	1.60	1.50	1.70	1.93	1.88	1.99	1.98	1.94	2.03	1.60	1.58	1.62
Adj. for education	1.70	1.60	1.81	2.14	2.07	2.20	2.11	2.06	2.16	1.62	1.60	1.64
Adj. for social class	1.64	1.54	1.74	1.91	1.85	1.97	1.93	1.89	1.98	1.65	1.63	1.68
Adj. for all SEP measures	1.37	1.28	1.46	1.69	1.64	1.75	1.83	1.79	1.88	1.55	1.53	1.58

**Figure 2:**
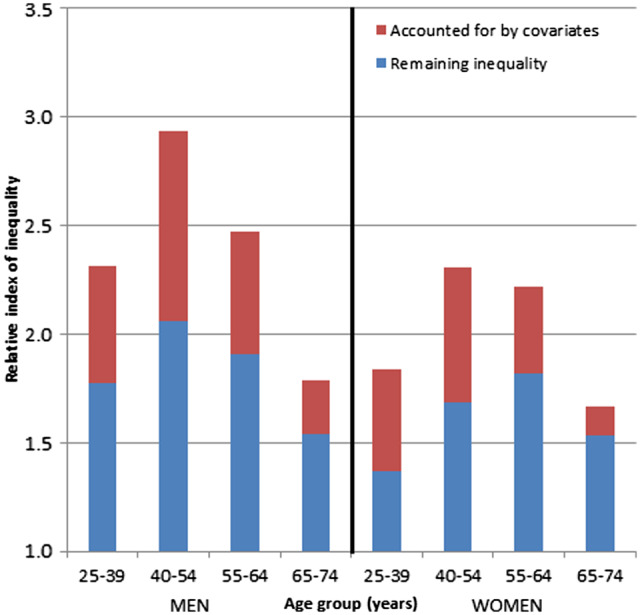
Relative indices of inequality (RII) in all-cause mortality by wealth, before and after adjustment for four measures of socio-economic position and other covariates in (a) men and (b) women. Each stacked bar indicates the extent that wealth is independently associated with all-cause mortality for different age–sex groups, before and after adjustment for covariates. Results for each age–sex group are from nested regression models, with RII = 1 indicating no association. Crude models adjusted for age (5-year bands) and follow-up; adjusted models additionally included education level, individual income, household income, occupational social class, number of children within household, region and country of birth. Coefficients for covariates are shown in the Supplementary Material, available as supplementary data at *IJE* online.

In adjusted analyses that accounted for all covariates including the four other measures of socio-economic position, substantial inequalities in mortality by wealth remained (Figure 2 and Supplementary Material pp 14–17). Adjustment accounted for less than half of wealth-based mortality inequalities for nearly all age-sex groups, except for women aged 25–39 years where adjustment attenuated the association slightly more. Substantial mortality inequalities by wealth were seen across all analyses.

**Figure 3:**
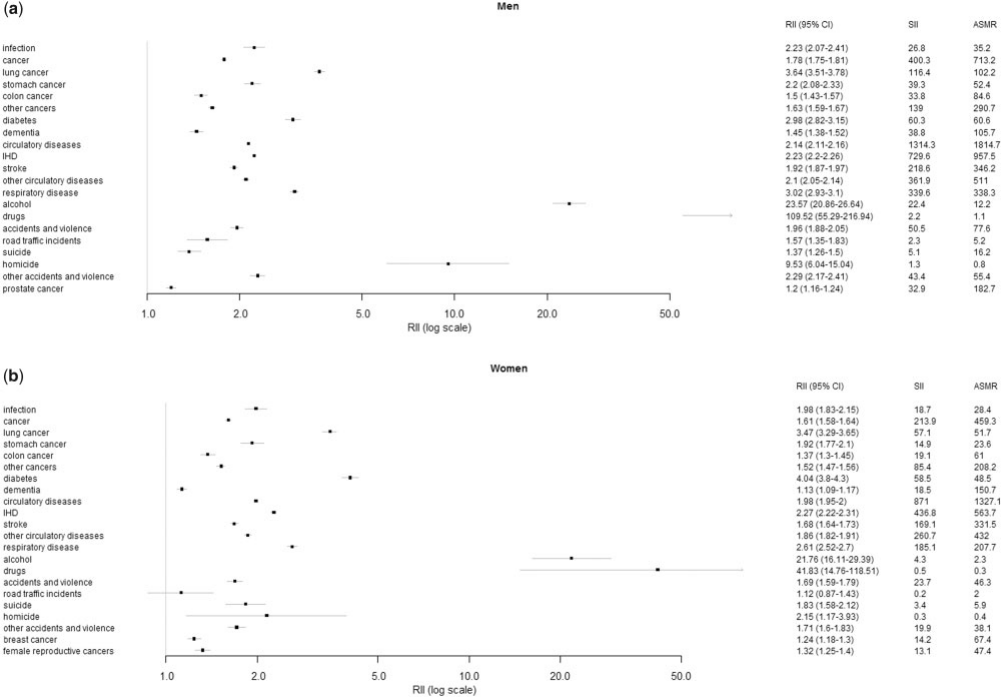
Relative indices of inequality (RII) in cause-specific mortality by wealth in Sweden among (a) men and (b) women. Analyses based on the entire Swedish population ≥25 years old. Coefficients are presented from separate regression models for men and women for each cause of death indicated. Error bars show 95% confidence intervals. Some causes of death are overlapping (e.g. cancer and lung cancer). International Classification of Disease codes used for classification and results for slope indices of inequality are provided in the Supplementary Material, available as supplementary data at *IJE* online.

The relative importance of different causes of mortality did not differ notably when assessing inequalities in mortality by wealth, individual income and household income (Supplementary Material pp 22–23). The greatest relative inequalities were seen for alcohol- and drug-related mortality in both men and women (Figure 3), with the magnitude of these associations being greater in men. However, circulatory diseases and cancers had the greatest age-standardized mortality rates and showed the largest absolute inequalities (indicated by the SIIs) in cause-specific mortality (Figure 4). Tobacco-related deaths were also likely to make an important contribution to inequalities, as indicated by the substantial increase in both relative and absolute risks of lung cancer and ischaemic heart disease. However, stroke-related mortality and the overall increased risk of cancers other than lung also suggests that improved health care could contribute to narrowing socio-economic inequalities.

When assessing wealth as a categorical variable, we found that lower wealth was consistently associated with greater all-cause mortality (Supplementary Material pp 18–21). As expected with this type of measure, the rate ratios between top and bottom quartiles were smaller than the RIIs. Adjustments for other measures of socio-economic position and other covariates again led to only modest attenuation of the association between wealth and mortality.

## Discussion

We examined all-cause and cause-specific inequalities in mortality by wealth for the whole adult population (aged ≥25 years) of Sweden, drawing on data from a historical wealth tax. Substantial wealth-based mortality inequalities throughout the adult life course and in both men and women were found. In comparison to assessments of individual income and household income, the patterning of mortality by wealth shows less fluctuation with age. For ages 25–74 years, adjustment for four other measures of socio-economic position only modestly attenuated the association. Comparison of inequalities in cause-specific mortality by wealth, individual income and household income showed similar causes of death were important across all three measures. Inequalities in non-communicable diseases, particularly cardiovascular diseases and cancers, were most important for population health and contributed the most to mortality inequalities.

Existing research on wealth and mortality is largely from survey-based studies, primarily in the USA and the UK^23–28^, although more recently extending across Europe and Taiwan.^29–31^ Overall, existing studies strongly echo our findings, demonstrating mortality inequalities by wealth. Furthermore, this relationship persisted after adjustment for other socio-economic measures such as education or income, and also after adjustment for health behaviours.^6^^,^^30^ More recently, research using the English Longitudinal Study of Ageing (ELSA) and the US Health and Retirement Study (HRS) have found similar inequalities for disability.^6^^,^^8^ Both of these studies were carried out in samples of older populations, whereas our study analysed the entire adult population. In comparison to the existing studies drawing on survey data, analyses of register data have been limited. Hoffmann analysed Danish register data for people >59 years old only and observed inequalities in mortality by wealth, although these were smaller than when assessed by income.^32^

Despite the large literature on the relationship between socio-economic position and mortality, studies focused on wealth have been rare. A frequent challenge is accessing appropriate data and therefore wealth related to housing has often been studied, typically demonstrating greater mortality among renters compared with home-owners and increased mortality among those living in smaller housing.^33–35^ Within Sweden, register data have been extensively used to study inequalities by income^36^, but rarely tried to distinguish potential wealth effects from income effects. By analysing annual income derived from capital (such as stocks, shares and savings accounts), Sabel *et al*. studied mortality inequalities and, in contrast to our results, found rates in young adults were higher among those with greater capital-derived income.^37^ However, both approaches do not capture overall wealth and the relative importance of different components of wealth (such as property or savings) may differ throughout the life course. By studying a comprehensive wealth indicator, we have been able to demonstrate that it is associated with mortality even in early adulthood.

Sweden is typically viewed as an egalitarian country, with social policies that redistribute income through relatively high taxation rates.^38^ Furthermore, the generosity of welfare policies in Nordic countries is thought to help buffer adverse health effects of economic hardship, in comparison to the more neoliberal policy approach of countries like the UK and the USA.^39–41^ Inequalities may be even greater elsewhere.

Our study has several strengths. First, it benefited from the unique availability of data from a historical wealth tax which provided a comprehensive picture of wealth that is likely to have greater validity than typically used survey measures. Second, we have explicitly compared wealth with other measures of material resources. Third, we have analysed high-quality register data, subject to minimal missing data. The use of data linkage has provided a large sample size, with complete follow-up. Analysing data for the whole adult population has allowed us to stratify our analyses and therefore report mortality inequalities by wealth for the whole adult life course.

However, limitations exist. Since the indicator was wealth tax paid, rather than wealth itself, we were unable to discriminate between people’s wealth below the wealth tax threshold. Furthermore, it is possible that some individuals kept wealth off-shore. To counter this issue, we utilized the RII measure that is based on an individual’s relative position within a society and is therefore likely to be more robust to these potential misclassifications. Furthermore, we investigated an alternative approach to categorizing wealth and found similar results. Relatedly, wealth tax information was only available for the year 1990. It was therefore impossible to study changes in wealth over time, make comparisons between individual and household wealth, or to compare wealth-based mortality inequalities over time. Third, we have been unable to investigate the role of specific behavioural risk factors, but these are likely to mediate the relationship between wealth and health rather than primarily confound it. Lastly, we note that our study reports only associations rather than estimating causal effects—further work is needed to establish causation, with natural experiment designs being potentially particularly helpful.^42^^,^^43^ The existence of historical or current wealth taxes in other countries with high-quality register data (such as Finland, Norway and Iceland) may provide opportunities for such research, as well as potential international comparative studies. Although data linkage is an important approach to addressing this gap, particularly if longitudinal wealth measures can be found, administrative and governance challenges may mean that conducting these studies is difficult.

Further work is needed to understand the mechanisms by which wealth may impact health. In contrast to income, the most frequently used measure of material resources, wealth accumulates across the life course. It is therefore often suggested that wealth may act as a resource that aids investment in health,^44^ with its cumulative nature reflecting prior socio-economic conditions and therefore opportunities for previous health investments.^28^ Wealth is also subject to less fluctuation than income and this raises the possibility that it is wealth’s ability to protect against economic insecurity that may be important.^45^ Given the accumulated nature of wealth and its relative permanence, it is often viewed as a preferable measure of socio-economic position in the elderly, but often not considered relevant at earlier ages. However, our finding of lower mortality among wealthier individuals in earlier adulthood (ages 25–34 years) suggests economic security may be an important pathway.

The increasing concentration of wealth across many societies appears likely to exacerbate health inequalities, with these inequalities occurring in young and older adults. Given that accumulation through earned income appears unlikely to give rise to substantial variations in wealth at early ages, this suggests that intergenerational transfers of wealth are important in perpetuating health inequalities. Efforts to bring about more progressive income taxation may therefore be inadequate to address health inequalities,^46^ with consideration for policies to narrow wealth inequalities needed.

## Supplementary Data

Supplementary data are available at *IJE* online.

## Funding

This work was supported by a NHS Research Scotland Senior Clinical Fellowship (SCAF/15/02), awarded to S.V.K.. S.V.K., R.D. and A.H.L. also acknowledge funding from the Medical Research Council (MC_UU_12017/13 & MC_UU_12017/15) and Scottish Government Chief Scientist Office (SPHSU13 & SPHSU15). C.L.N. was supported by a Medical Research Council fellowship (MR/R024774/1).

We would also like to acknowledge Reidar **Ö**sterman for extracting the datasets and explaining the derivation of key variables, Desmond Campbell for assistance with creating figures in R and Audrey Hendry for assistance with formatting the manuscript.

## Author Contributions

S.V.K. conceived the idea for the study, conducted the analysis and drafted the manuscript. All authors contributed to the study design, interpretation of results and critically revised the manuscript. All authors approved the final version of the paper.

## Conflict of Interest

Except for the funding acknowledged above, we declare no competing interests.

## Supplementary Material

dyaa053_Supplementary_Data
