# Supplementary material for: Inequalities in all-cause and cause-specific mortality across the life course by wealth and income in Sweden: a register-based cohort study
Source: Int J Epidemiol. 2020 May 7;49(3):917–25. doi: 10.1093/ije/dyaa053 (PMC7394946; doi:10.1093/ije/dyaa053)
Supplement: dyaa053_Supplementary_Data [file dyaa053_supplementary_data.docx]

**Web Appendix**

**Contents**

1. Construction of the Relative Index of Inequality (RII) pg 2
2. ICD codes used to categorise cause-specific mortality, based on underlying cause of death pg 3
3. Study population included in main and adjusted analyses, stratified by age group and sex pg 4
4. Number and percentage of study population paying zero wealth tax, stratified by age group and sex pg 6
5. Descriptive characteristics of study population used in adjusted analyses, stratified by age group and sex pg 7
6. Relative Indices of Inequality (RII) by wealth, adjusting for four different measures of socioeconomic position, age group and follow-up period in men pg 8
7. Relative Indices of Inequality (RII) by wealth, adjusting for four different measures of socioeconomic position, age group and follow-up period in women pg 11
8. Relative Indices of Inequality (RII) by wealth in crude and fully adjusted models, stratified by age group and sex pg 14
9. Inequalities in all-cause mortality by wealth tax paid (assessed by a categorical variable), adjusted for age group and follow-up period pg 18
10. Inequalities in all-cause mortality by wealth tax paid (assessed by a categorical variable), adjusted for a) age group and follow-up period, b) four measures of socioeconomic position, and c) all covariates pg 19
11. Relative and slope indices of inequality for cause-specific mortality by wealth, individual income and household income (adjusted for age and follow-up period) pg 22

**1. Construction of the relative index of inequality**

To illustrate the calculation of the RII, see the below simplified diagram. In the simple situation where there are three categories for wealth, everyone can be considered ranked from the most advantaged to the least advantaged. The midpoint of the cumulative distribution is allocated for everyone in a specific group and this is the exposure used within a regression model to estimate the relative index of inequality (RII). For example, consider the below scenario where the highest wealth group contains 20% of the population, the medium group 30% and the lowest group 50%. The standardised rank for the lowest group is 0.1 (midpoint of 20%); for the middle group is 0.35 (0.2+midpoint of 30%); and for the lowest group is 0.75 (0.5+midpoint of 50%).

This approach can be generalised to create a continuous rank-based measure, with in our case, a proportion of the population having the same rank (due to not paying any wealth tax).

More details about the RII can be found here:

Mackenbach JP, Kunst AE. Measuring the magnitude of socio-economic inequalities in health: An overview of available measures illustrated with two examples from Europe. Soc Sci Med. 1997;44(6):757-71.

**2. ICD codes used to categorise cause-specific mortality, based on underlying cause of death**

| **Category** | **ICD 9 codes** | **ICD 10 codes** |
| --- | --- | --- |
| Infection | 001-018, 020-066, 070-088, 090-134, 136-139 | A00-A09, A15-A99, B00-B39, G00, G01, G02 |
| Cancers | 140-208 | C |
| Lung cancer | 162 | C33 C34 |
| Breast cancer | 174, 175 | C50 |
| Prostate cancer | 185 | C61 |
| Female reproductive cancer | 179-183 | C53-C56 |
| Stomach and oesophagus cancer | 150, 151 | C15, C16 |
| Colorectal cancer | 153, 154 | C18-C21 |
| Other cancers | All cancers (140-208) not listed in six above specific cancer categories | All cancers (C) not listed in six above specific cancer categories |
| Diabetes | 250 | E10-E14 |
| Dementias | 290, 331 | F01, F02, F03, G30, G31.9, G31.1, G31.83 |
| Circulatory disease | 401-404, 410-438, 440-448 | I00- I09, I11, I13, I2, I3, I4, I50-I51, I60-I69, I70-I79 |
| Ischaemic heart disease | 410-414, 4292 | I21-I25 |
| Stroke | 430-438 | I60-I69 |
| Other circulatory diseases | All circulatory disease codes not listed in two above specific categories | All circulatory disease codes not listed in two above specific categories |
| Respiratory diseases | 460-466, 470-478, 480-488, 490-496, 500-519 | J0, J10-J18, J2, J3, J40-J47, J60-J68, J7, J8, J9 |
| Alcohol-related diseases | 291, 303, 3050 | F10 |
| Drug-related diseases | 304, 3052-3059 | F11-F16, F18, F19 |
| Accidents and violence | E800-E807, E820-E829, E83, E841-E848, E87, E88, E89, E90, E91, E920-E929, E95, E96, | V, W, X, Y, U01-U03 |
| Road traffic incidents | E81, E20-E825 | V02, V03, V04, V090-V093, V12, V13, V14, V190-V199, V2-V7, V803-V805, V810, V811, V820, V821, V83-V87, V880-V888, V890, V892, V980 |
| Suicides | E95 | X6, X7, X80-X84, Y780, U03 |
| Homicides | E96 | X85-X89, X9, Y0, Y871, U01, U02 |
| Other accidents or violence | All accidents and violence codes not listed in three above specific categories | All accidents and violence codes not listed in three above specific categories |

**3. Study population included in main and adjusted analyses, stratified by age group and sex**

| **MEN** | **25-39 years** | | | **40-54 years** | | | **55-64 years** | | | **65-74 years** | | |
| --- | --- | --- | --- | --- | --- | --- | --- | --- | --- | --- | --- | --- |
|  | N | PYrs | Deaths | N | PYrs | Deaths | N | PYrs | Deaths | N | PYrs | Deaths |
| Sample included in main analysis | 952,005 | 17,165,730 | 28,794 | 878,514 | 15,710,162 | 99,581 | 413,735 | 6,672,941 | 150,155 | 398,024 | 5,092,633 | 288,319 |
| Excluded from main analysis due to missing income/wealth data | 90 | 1543 | 5 | 26 | 456 | 1 | 14 | 251 | 1 | 2 | 15 | 1 |
| Percentage excluded from main analysis | 0.0095 | 0.0090 | 0.0174 | 0.0030 | 0.0029 | 0.0010 | 0.0034 | 0.0038 | 0.0007 | 0.0005 | 0.0003 | 0.0003 |
| Sample included in adjusted analyses | 871,384 | 15,924,419 | 26,165 | 850,923 | 15,329,924 | 94,203 | 400,679 | 6,543,874 | 142,750 | 372,779 | 4,918,600 | 267,101 |
| Excluded from adjusted analyses due to missing covariates | 80,566 | 1,241,311 | 2,629 | 27,591 | 380,238 | 5,378 | 13,056 | 129,067 | 7,405 | 25,245 | 174,033 | 21,218 |
| Percentage excluded from adjusted analyses | 8.47 | 7.24 | 9.15 | 3.14 | 2.42 | 5.40 | 3.16 | 1.94 | 4.93 | 6.34 | 3.42 | 7.36 |
| Total population | 952,095 | 17,167,273 | 28,799 | 878,540 | 15,710,618 | 99,582 | 413,749 | 6,673,192 | 150,156 | 398,026 | 5,092,648 | 288,320 |

| **MEN** | **75-84 years** | | | **85+ years** | | |
| --- | --- | --- | --- | --- | --- | --- |
|  | N | PYrs | Deaths | N | PYrs | Deaths |
| Sample included in main analysis | 235,605 | 1,901,622 | 227,400 | 55,577 | 255,559 | 55,213 |
| Excluded from main analysis due to missing income/wealth data | 0 | 0 | 0 | 0 | 0 | 0 |
| Percentage excluded from main analysis | 0 | 0 | 0 | 0 | 0 | 0 |
| Total population | 235,605 | 1,901,622 | 227,400 | 55,577 | 255,559 | 55,213 |

N=number of participants; PYrs=number of person-years.

| **WOMEN** | **25-39 years** | | | **40-54 years** | | | **55-64 years** | | | **65-74 years** | | |
| --- | --- | --- | --- | --- | --- | --- | --- | --- | --- | --- | --- | --- |
|  | N | PYrs | Deaths | N | PYrs | Deaths | N | PYrs | Deaths | N | PYrs | Deaths |
| Sample included in main analysis | 902,505 | 16,485,965 | 16,887 | 850,416 | 15,533,697 | 63,350 | 436,795 | 7,543,779 | 101,854 | 459,206 | 6,798,986 | 262,505 |
| Excluded from main analysis due to missing income/wealth data | 64 | 1,107 | 0 | 17 | 280 | 1 | 25 | 454 | 3 | 6 | 104 | 2 |
| Percentage excluded from main analysis | 0.0071 | 0.0067 | 0.0000 | 0.0020 | 0.0018 | 0.0016 | 0.0057 | 0.0060 | 0.0029 | 0.0013 | 0.0015 | 0.0008 |
| Sample included in adjusted analyses | 833,443 | 15,372,442 | 15,437 | 826,893 | 15,178,771 | 60,253 | 423,521 | 7,371,071 | 97,018 | 434,339 | 6,542,964 | 245,880 |
| Excluded from adjusted analyses due to missing covariates | 69,062 | 1,113,523 | 14,450 | 23,523 | 354,926 | 3,097 | 13,274 | 172,708 | 4,836 | 24,867 | 256,022 | 16,625 |
| Percentage excluded from adjusted analyses | 7.66 | 6.76 | 85.57 | 2.77 | 2.29 | 4.89 | 3.04 | 2.30 | 4.75 | 5.42 | 3.77 | 6.33 |
| Total population | 902,569 | 16,487,072 | 16,887 | 850,433 | 15,533,977 | 63,351 | 436,820 | 7,544,233 | 101,857 | 459,212 | 6,799,090 | 262,507 |

| **WOMEN** | **75-84 years** | | | **85+ years** | | |
| --- | --- | --- | --- | --- | --- | --- |
|  | N | Pyrs | Deaths | N | Pyrs | Deaths |
| Sample included in main analysis | 338,688 | 3,344,384 | 313,216 | 121,259 | 649,093 | 120,639 |
| Excluded from main analysis due to missing income/wealth data | 2 | 14 | 2 | 0 | 0 | 0 |
| Percentage excluded from main analysis | 0.0006 | 0.0004 | 0.0006 | 0 | 0 | 0 |
| Total population | 338,690 | 3,344,398 | 313,218 | 121,259 | 649,093 | 120,639 |

N=number of participants; PYrs=number of person-years.

**4. Number and percentage of study population paying zero wealth tax, stratified by age group and sex**

|  |  | **Age group** | | | | | |  |
| --- | --- | --- | --- | --- | --- | --- | --- | --- |
|  |  | 25-39 | 40-54 | 55-64 | 65-74 | 75-84 | 85+ | Total |
| **Men** | Number | 550,006 | 359,034 | 98,386 | 52,623 | 24,065 | 5,625 | 1,089,739 |
|  | % | 61.4 | 41.5 | 24.1 | 13.3 | 10.2 | 10.2 | 38.2 |
| **Women** | Number | 455,852 | 280,732 | 90,829 | 61,304 | 44,429 | 17,306 | 950,452 |
|  | % | 53.7 | 33.6 | 21.2 | 13.4 | 13.2 | 14.3 | 31.4 |

**5. Descriptive characteristics of study population used in adjusted analyses, stratified by age group and sex**

|  | MEN | | | | WOMEN | | | |
| --- | --- | --- | --- | --- | --- | --- | --- | --- |
|  | 25-39 | 40-54 | 55-64 | 65-74 | 25-39 | 40-54 | 55-64 | 65-74 |
| **Education** |  |  |  |  |  |  |  |  |
| Degree or higher | 216,314 | 193,636 | 58,336 | 36,503 | 229,352 | 207,153 | 53,241 | 26,110 |
| Upper secondary | 456,370 | 347,722 | 126,248 | 103,772 | 447,742 | 342,533 | 127,584 | 100,483 |
| Compulsory only | 198,700 | 309,526 | 216,059 | 232,490 | 156,323 | 277,188 | 242,669 | 307,732 |
| **Social class** |  |  |  |  |  |  |  |  |
| Upper non-manual | 92,165 | 136,905 | 44,734 | 5,539 | 57,154 | 72,918 | 19,162 | 1,256 |
| Intermediate non-manual | 127,500 | 150,899 | 51,277 | 3,798 | 147,232 | 146,545 | 38,980 | 1,840 |
| Lower non-manual | 73,158 | 75,038 | 34,886 | 2,704 | 143,683 | 169,119 | 62,130 | 3,278 |
| Skilled manual | 189,351 | 152,203 | 52,178 | 2,110 | 97,750 | 65,823 | 17,790 | 545 |
| Unskilled manual | 196,223 | 154,853 | 61,717 | 3,635 | 214,806 | 221,466 | 107,254 | 3,875 |
| Farmers and farm labourers | 11,188 | 17,588 | 13,595 | 6,733 | 3,278 | 7,580 | 5,554 | 1,203 |
| Self-employed | 36,103 | 57,798 | 23,063 | 7,057 | 16,846 | 27,491 | 9,410 | 2,347 |
| Unclassified employee | 61,708 | 37,783 | 17,979 | 25,400 | 51,785 | 28,952 | 15,735 | 13,168 |
| Economically inactive | 83,988 | 67,817 | 101,214 | 315,789 | 100,883 | 86,980 | 147,479 | 406,813 |
| **No. of adults in household** |  |  |  |  |  |  |  |  |
| None | 787,975 | 602,926 | 321,636 | 342,652 | 784,759 | 534,882 | 354,720 | 405,248 |
| 1 adult | 62,671 | 202,552 | 64,360 | 26,700 | 40,255 | 235,742 | 58,309 | 26,883 |
| 2+ adults | 20,738 | 45,406 | 14,647 | 3,413 | 8,403 | 56,250 | 10,465 | 2,194 |
| **No. of children in household** |  |  |  |  |  |  |  |  |
| None | 453,092 | 452,082 | 376,286 | 370,333 | 258,783 | 464,587 | 416,609 | 434,230 |
| 1 child | 134,599 | 182,145 | 18,761 | 1,917 | 181,891 | 206,399 | 6,360 | 88 |
| 2-3 children | 266,595 | 203,694 | 5,288 | 484 | 367,440 | 148,918 | 514 | 7 |
| 4+ children | 17,098 | 12,963 | 308 | 31 | 25,303 | 6,970 | 11 | 0 |
| **Country of birth** |  |  |  |  |  |  |  |  |
| Born in Sweden | 774,056 | 760,028 | 361,105 | 352,848 | 736,366 | 734,677 | 373,569 | 404,965 |
| Born abroad | 97,328 | 90,856 | 39,538 | 19,917 | 97,051 | 92,197 | 49,925 | 29,360 |

**6. Relative Indices of Inequality (RII) by wealth, adjusting for four different measures of socioeconomic position, age group and follow-up period in men**

| **MEN** | **25-39 years** | | | | **40-54 years** | | | | **55-64 years** | | | | **65-74 years** | | | |
| --- | --- | --- | --- | --- | --- | --- | --- | --- | --- | --- | --- | --- | --- | --- | --- | --- |
|  | **IRR** | **P** | **L 95** | **U 95** | **IRR** | **P** | **L 95** | **U 95** | **IRR** | **P** | **L 95** | **U 95** | **IRR** | **P** | **L 95** | **U 95** |
| **Crude model** |  |  |  |  |  |  |  |  |  |  |  |  |  |  |  |  |
| Wealth RII | **2.32** | <0.001 | 2.20 | 2.44 | **2.95** | <0.001 | 2.88 | 3.02 | **2.50** | <0.001 | 2.45 | 2.55 | **1.82** | <0.001 | 1.80 | 1.84 |
|  |  |  |  |  |  |  |  |  |  |  |  |  |  |  |  |  |
| **Adj. for income** |  |  |  |  |  |  |  |  |  |  |  |  |  |  |  |  |
| Wealth RII | **2.17** | <0.001 | 2.06 | 2.29 | **2.58** | <0.001 | 2.51 | 2.64 | **2.24** | <0.001 | 2.20 | 2.28 | **1.64** | <0.001 | 1.62 | 1.67 |
|  |  |  |  |  |  |  |  |  |  |  |  |  |  |  |  |  |
| *Individual income* |  |  |  |  |  |  |  |  |  |  |  |  |  |  |  |  |
| 2 | **1.17** | <0.001 | 1.12 | 1.23 | **1.18** | <0.001 | 1.16 | 1.21 | **1.16** | <0.001 | 1.13 | 1.18 | **1.17** | <0.001 | 1.15 | 1.18 |
| 3 | **1.46** | <0.001 | 1.40 | 1.53 | **1.39** | <0.001 | 1.35 | 1.42 | **1.31** | <0.001 | 1.29 | 1.34 | **1.24** | <0.001 | 1.22 | 1.26 |
| 4 | **2.07** | <0.001 | 1.98 | 2.16 | **1.73** | <0.001 | 1.69 | 1.77 | **1.57** | <0.001 | 1.54 | 1.60 | **1.32** | <0.001 | 1.30 | 1.33 |
| Lowest quintile | **3.51** | <0.001 | 3.37 | 3.65 | **2.77** | <0.001 | 2.71 | 2.83 | **1.84** | <0.001 | 1.80 | 1.87 | **1.44** | <0.001 | 1.42 | 1.46 |
|  |  |  |  |  |  |  |  |  |  |  |  |  |  |  |  |  |
| **Adj. for household income** |  |  |  |  |  |  |  |  |  |  |  |  |  |  |  |  |
| Wealth RII | **2.14** | <0.001 | 2.03 | 2.25 | **2.42** | <0.001 | 2.36 | 2.48 | **2.16** | <0.001 | 2.11 | 2.20 | **1.64** | <0.001 | 1.62 | 1.66 |
|  |  |  |  |  |  |  |  |  |  |  |  |  |  |  |  |  |
| *Household income* |  |  |  |  |  |  |  |  |  |  |  |  |  |  |  |  |
| 2 | **0.96** | 0.049 | 0.92 | 1.00 | **1.10** | <0.001 | 1.07 | 1.13 | **1.12** | <0.001 | 1.10 | 1.14 | **1.09** | <0.001 | 1.08 | 1.10 |
| 3 | **1.28** | <0.001 | 1.23 | 1.34 | **1.26** | <0.001 | 1.23 | 1.29 | **1.26** | <0.001 | 1.24 | 1.28 | **1.17** | <0.001 | 1.15 | 1.18 |
| 4 | **1.55** | <0.001 | 1.49 | 1.62 | **1.60** | <0.001 | 1.57 | 1.64 | **1.41** | <0.001 | 1.39 | 1.44 | **1.26** | <0.001 | 1.24 | 1.27 |
| Lowest quintile | **2.95** | <0.001 | 2.84 | 3.07 | **2.84** | <0.001 | 2.78 | 2.90 | **2.02** | <0.001 | 1.98 | 2.05 | **1.54** | <0.001 | 1.52 | 1.56 |
|  |  |  |  |  |  |  |  |  |  |  |  |  |  |  |  |  |
| **Adj. for education** |  |  |  |  |  |  |  |  |  |  |  |  |  |  |  |  |
| Wealth RII | **2.16** | <0.001 | 2.05 | 2.27 | **2.84** | <0.001 | 2.77 | 2.91 | **2.42** | <0.001 | 2.37 | 2.46 | **1.76** | <0.001 | 1.74 | 1.79 |
|  |  |  |  |  |  |  |  |  |  |  |  |  |  |  |  |  |
| *Education* |  |  |  |  |  |  |  |  |  |  |  |  |  |  |  |  |
| Upper secondary | **1.80** | <0.001 | 1.74 | 1.87 | **1.47** | <0.001 | 1.44 | 1.50 | **1.23** | <0.001 | 1.21 | 1.25 | **1.15** | <0.001 | 1.14 | 1.17 |
| Compulsory only | **2.58** | <0.001 | 2.48 | 2.68 | **1.79** | <0.001 | 1.75 | 1.82 | **1.43** | <0.001 | 1.41 | 1.46 | **1.31** | <0.001 | 1.29 | 1.33 |

RII=relative index of inequality; P=p value; L 95=lower 95% confidence interval; U95=upper 95% confidence interval

| **MEN** | **25-39 years** | | | | **40-54 years** | | | | **55-64 years** | | | | **65-74 years** | | | |
| --- | --- | --- | --- | --- | --- | --- | --- | --- | --- | --- | --- | --- | --- | --- | --- | --- |
|  | **IRR** | **P** | **L 95** | **U 95** | **IRR** | **P** | **L 95** | **U 95** | **IRR** | **P** | **L 95** | **U 95** | **IRR** | **P** | **L 95** | **U 95** |
| **Adj. for social class** |  |  |  |  |  |  |  |  |  |  |  |  |  |  |  |  |
| Wealth RII | **1.90** | <0.001 | 1.81 | 2.01 | **2.35** | <0.001 | 2.29 | 2.41 | **2.14** | <0.001 | 2.10 | 2.18 | **1.76** | <0.001 | 1.73 | 1.78 |
|  |  |  |  |  |  |  |  |  |  |  |  |  |  |  |  |  |
| *Social class* |  |  |  |  |  |  |  |  |  |  |  |  |  |  |  |  |
| Intermediate non-manual | **1.23** | <0.001 | 1.16 | 1.32 | **1.16** | <0.001 | 1.13 | 1.19 | **1.08** | <0.001 | 1.05 | 1.11 | **1.03** | 0.31 | 0.97 | 1.09 |
| Lower non-manual | **1.45** | <0.001 | 1.35 | 1.55 | **1.45** | <0.001 | 1.41 | 1.49 | **1.22** | <0.001 | 1.19 | 1.26 | **1.11** | 0.001 | 1.04 | 1.18 |
| Skilled manual | **1.74** | <0.001 | 1.64 | 1.84 | **1.56** | <0.001 | 1.53 | 1.60 | **1.25** | <0.001 | 1.22 | 1.28 | **1.13** | <0.001 | 1.06 | 1.21 |
| Unskilled manual | **2.09** | <0.001 | 1.97 | 2.21 | **1.77** | <0.001 | 1.73 | 1.82 | **1.37** | <0.001 | 1.34 | 1.40 | **1.18** | <0.001 | 1.12 | 1.25 |
| Farmers and farm labourers | **1.67** | <0.001 | 1.47 | 1.89 | **1.29** | <0.001 | 1.21 | 1.36 | **1.18** | <0.001 | 1.13 | 1.22 | **1.33** | <0.001 | 1.27 | 1.40 |
| Self-employed | **1.59** | <0.001 | 1.47 | 1.72 | **1.44** | <0.001 | 1.39 | 1.49 | **1.25** | <0.001 | 1.21 | 1.29 | **1.29** | <0.001 | 1.23 | 1.35 |
| Unclassified employees | **2.85** | <0.001 | 2.67 | 3.04 | **2.19** | <0.001 | 2.12 | 2.26 | **1.64** | <0.001 | 1.59 | 1.69 | **1.34** | <0.001 | 1.29 | 1.40 |
| Economically inactive | **5.36** | <0.001 | 5.06 | 5.67 | **3.71** | <0.001 | 3.62 | 3.80 | **2.10** | <0.001 | 2.05 | 2.14 | **1.59** | <0.001 | 1.53 | 1.65 |

RII=relative index of inequality; P=p value; L 95=lower 95% confidence interval; U95=upper 95% confidence interval

| **MEN** | **25-39 years** | | | | **40-54 years** | | | | **55-64 years** | | | | **65-74 years** | | | |
| --- | --- | --- | --- | --- | --- | --- | --- | --- | --- | --- | --- | --- | --- | --- | --- | --- |
|  | **IRR** | **P** | **L 95** | **U 95** | **IRR** | **P** | **L 95** | **U 95** | **IRR** | **P** | **L 95** | **U 95** | **IRR** | **P** | **L 95** | **U 95** |
| **Adj. for all SEP measures** |  |  |  |  |  |  |  |  |  |  |  |  |  |  |  |  |
| Wealth RII | **1.78** | <0.001 | 1.69 | 1.88 | **2.07** | <0.001 | 2.02 | 2.12 | **1.93** | <0.001 | 1.89 | 1.96 | **1.56** | <0.001 | 1.54 | 1.59 |
|  |  |  |  |  |  |  |  |  |  |  |  |  |  |  |  |  |
| *Individual income* |  |  |  |  |  |  |  |  |  |  |  |  |  |  |  |  |
| 2 | **0.99** | 0.78 | 0.94 | 1.04 | **1.06** | <0.001 | 1.04 | 1.09 | **1.05** | <0.001 | 1.03 | 1.07 | **1.05** | <0.001 | 1.04 | 1.07 |
| 3 | **1.15** | <0.001 | 1.09 | 1.21 | **1.06** | <0.001 | 1.03 | 1.09 | **1.06** | <0.001 | 1.04 | 1.08 | **1.03** | <0.001 | 1.01 | 1.04 |
| 4 | **1.36** | <0.001 | 1.29 | 1.43 | **1.14** | <0.001 | 1.10 | 1.17 | **1.11** | <0.001 | 1.08 | 1.13 | **1.06** | <0.001 | 1.04 | 1.07 |
| Lowest quintile | **1.69** | <0.001 | 1.60 | 1.79 | **1.42** | <0.001 | 1.38 | 1.47 | **1.15** | <0.001 | 1.12 | 1.18 | **1.13** | <0.001 | 1.11 | 1.15 |
|  |  |  |  |  |  |  |  |  |  |  |  |  |  |  |  |  |
| *Household income* |  |  |  |  |  |  |  |  |  |  |  |  |  |  |  |  |
| 2 | **0.85** | <0.001 | 0.81 | 0.89 | **1.02** | 0.211 | 0.99 | 1.04 | **1.06** | <0.001 | 1.04 | 1.08 | **1.02** | <0.001 | 1.01 | 1.04 |
| 3 | **0.97** | 0.158 | 0.92 | 1.01 | **1.08** | <0.001 | 1.05 | 1.11 | **1.12** | <0.001 | 1.09 | 1.14 | **1.07** | <0.001 | 1.05 | 1.08 |
| 4 | **1.24** | <0.001 | 1.18 | 1.30 | **1.28** | <0.001 | 1.25 | 1.31 | **1.21** | <0.001 | 1.18 | 1.23 | **1.12** | <0.001 | 1.11 | 1.14 |
| Lowest quintile | **1.46** | <0.001 | 1.38 | 1.53 | **1.88** | <0.001 | 1.83 | 1.93 | **1.60** | <0.001 | 1.56 | 1.63 | **1.36** | <0.001 | 1.34 | 1.38 |
|  |  |  |  |  |  |  |  |  |  |  |  |  |  |  |  |  |
| *Education* |  |  |  |  |  |  |  |  |  |  |  |  |  |  |  |  |
| Upper secondary | **1.56** | <0.001 | 1.50 | 1.63 | **1.24** | <0.001 | 1.21 | 1.27 | **1.07** | <0.001 | 1.04 | 1.09 | **1.07** | <0.001 | 1.05 | 1.09 |
| Compulsory only | **2.01** | <0.001 | 1.93 | 2.10 | **1.33** | <0.001 | 1.30 | 1.36 | **1.12** | <0.001 | 1.10 | 1.14 | **1.13** | <0.001 | 1.12 | 1.15 |
|  |  |  |  |  |  |  |  |  |  |  |  |  |  |  |  |  |
| *Social class* |  |  |  |  |  |  |  |  |  |  |  |  |  |  |  |  |
| Intermediate non-manual | **1.06** | 0.084 | 0.99 | 1.13 | **1.00** | 0.94 | 0.97 | 1.03 | **0.99** | 0.575 | 0.97 | 1.02 | **0.99** | 0.814 | 0.94 | 1.05 |
| Lower non-manual | **0.99** | 0.783 | 0.92 | 1.07 | **1.08** | <0.001 | 1.04 | 1.11 | **1.03** | 0.029 | 1.00 | 1.06 | **1.04** | 0.271 | 0.97 | 1.10 |
| Skilled manual | **1.08** | 0.013 | 1.02 | 1.16 | **1.07** | <0.001 | 1.04 | 1.10 | **1.01** | 0.352 | 0.99 | 1.04 | **1.00** | 0.978 | 0.93 | 1.07 |
| Unskilled manual | **1.16** | <0.001 | 1.09 | 1.24 | **1.11** | <0.001 | 1.07 | 1.14 | **1.05** | 0.001 | 1.02 | 1.08 | **1.05** | 0.097 | 0.99 | 1.11 |
| Farmers and farm labourers | **0.67** | <0.001 | 0.58 | 0.76 | **0.63** | <0.001 | 0.59 | 0.67 | **0.79** | <0.001 | 0.76 | 0.83 | **1.00** | 0.896 | 0.95 | 1.05 |
| Self-employed | **0.79** | <0.001 | 0.72 | 0.86 | **0.87** | <0.001 | 0.83 | 0.90 | **0.95** | 0.001 | 0.92 | 0.98 | **1.11** | <0.001 | 1.05 | 1.16 |
| Unclassified employees | **1.45** | <0.001 | 1.36 | 1.56 | **1.34** | <0.001 | 1.29 | 1.39 | **1.29** | <0.001 | 1.25 | 1.34 | **1.12** | <0.001 | 1.08 | 1.17 |
| Economically inactive | **2.25** | <0.001 | 2.11 | 2.40 | **1.87** | <0.001 | 1.81 | 1.93 | **1.53** | <0.001 | 1.49 | 1.57 | **1.31** | <0.001 | 1.26 | 1.36 |

RII=relative index of inequality; P=p value; L 95=lower 95% confidence interval; U95=upper 95% confidence interval

**7. Relative Indices of Inequality (RII) by wealth, adjusting for four different measures of socioeconomic position, age group and follow-up period in women**

| **WOMEN** | **25-39 years** | | | | **40-54 years** | | | | **55-64 years** | | | | **65-74 years** | | | |
| --- | --- | --- | --- | --- | --- | --- | --- | --- | --- | --- | --- | --- | --- | --- | --- | --- |
|  | **IRR** | **P** | **L 95** | **U 95** | **IRR** | **P** | **L 95** | **U 95** | **IRR** | **P** | **L 95** | **U 95** | **IRR** | **P** | **L 95** | **U 95** |
| **Crude model** |  |  |  |  |  |  |  |  |  |  |  |  |  |  |  |  |
| Wealth | **1.84** | <0.001 | 1.73 | 1.95 | **2.31** | <0.001 | 2.24 | 2.38 | **2.24** | <0.001 | 2.19 | 2.29 | **1.69** | <0.001 | 1.67 | 1.71 |
|  |  |  |  |  |  |  |  |  |  |  |  |  |  |  |  |  |
| **Adj. for income** |  |  |  |  |  |  |  |  |  |  |  |  |  |  |  |  |
| Wealth | **1.90** | <0.001 | 1.79 | 2.03 | **2.29** | <0.001 | 2.22 | 2.36 | **2.15** | <0.001 | 2.10 | 2.20 | **1.66** | <0.001 | 1.63 | 1.68 |
| *Individual income* |  |  |  |  |  |  |  |  |  |  |  |  |  |  |  |  |
| 2 | **1.00** | 0.941 | 0.95 | 1.05 | **1.08** | <0.001 | 1.05 | 1.12 | **1.10** | <0.001 | 1.08 | 1.13 | **1.11** | <0.001 | 1.10 | 1.13 |
| 3 | **1.06** | 0.028 | 1.01 | 1.11 | **1.17** | <0.001 | 1.14 | 1.20 | **1.24** | <0.001 | 1.21 | 1.26 | **1.15** | <0.001 | 1.14 | 1.17 |
| 4 | **1.17** | <0.001 | 1.11 | 1.23 | **1.32** | <0.001 | 1.28 | 1.35 | **1.31** | <0.001 | 1.28 | 1.34 | **1.07** | <0.001 | 1.06 | 1.09 |
| Lowest quintile | **1.35** | <0.001 | 1.29 | 1.42 | **1.49** | <0.001 | 1.45 | 1.53 | **1.23** | <0.001 | 1.20 | 1.25 | **1.00** | 0.979 | 0.99 | 1.01 |
|  |  |  |  |  |  |  |  |  |  |  |  |  |  |  |  |  |
| **Adj. for household income** |  |  |  |  |  |  |  |  |  |  |  |  |  |  |  |  |
| Wealth | **1.60** | <0.001 | 1.50 | 1.70 | **1.93** | <0.001 | 1.88 | 1.99 | **1.98** | <0.001 | 1.94 | 2.03 | **1.60** | <0.001 | 1.58 | 1.62 |
| *Household income* |  |  |  |  |  |  |  |  |  |  |  |  |  |  |  |  |
| 2 | **1.02** | 0.418 | 0.97 | 1.08 | **1.09** | <0.001 | 1.06 | 1.13 | **1.12** | <0.001 | 1.09 | 1.14 | **1.08** | <0.001 | 1.07 | 1.10 |
| 3 | **1.20** | <0.001 | 1.14 | 1.27 | **1.26** | <0.001 | 1.22 | 1.30 | **1.25** | <0.001 | 1.22 | 1.28 | **1.16** | <0.001 | 1.14 | 1.18 |
| 4 | **1.47** | <0.001 | 1.40 | 1.55 | **1.48** | <0.001 | 1.44 | 1.52 | **1.39** | <0.001 | 1.36 | 1.42 | **1.26** | <0.001 | 1.25 | 1.28 |
| Lowest quintile | **2.19** | <0.001 | 2.08 | 2.30 | **2.03** | <0.001 | 1.98 | 2.08 | **1.75** | <0.001 | 1.72 | 1.79 | **1.41** | <0.001 | 1.39 | 1.43 |
|  |  |  |  |  |  |  |  |  |  |  |  |  |  |  |  |  |
| **Adj. for education** |  |  |  |  |  |  |  |  |  |  |  |  |  |  |  |  |
| Wealth | **1.70** | <0.001 | 1.60 | 1.81 | **2.14** | <0.001 | 2.07 | 2.20 | **2.11** | <0.001 | 2.06 | 2.16 | **1.62** | <0.001 | 1.60 | 1.64 |
|  |  |  |  |  |  |  |  |  |  |  |  |  |  |  |  |  |
| *Education* |  |  |  |  |  |  |  |  |  |  |  |  |  |  |  |  |
| Upper secondary | **1.41** | <0.001 | 1.36 | 1.47 | **1.33** | <0.001 | 1.30 | 1.36 | **1.21** | <0.001 | 1.18 | 1.24 | **1.16** | <0.001 | 1.13 | 1.18 |
| Compulsory only | **2.19** | <0.001 | 2.09 | 2.29 | **1.64** | <0.001 | 1.60 | 1.67 | **1.43** | <0.001 | 1.40 | 1.47 | **1.31** | <0.001 | 1.29 | 1.34 |

RII=relative index of inequality; P=p value; L 95=lower 95% confidence interval; U95=upper 95% confidence interval

| **WOMEN** | **25-39 years** | | | | **40-54 years** | | | | **55-64 years** | | | | **65-74 years** | | | |
| --- | --- | --- | --- | --- | --- | --- | --- | --- | --- | --- | --- | --- | --- | --- | --- | --- |
|  | **IRR** | **P** | **L 95** | **U 95** | **IRR** | **P** | **L 95** | **U 95** | **IRR** | **P** | **L 95** | **U 95** | **IRR** | **P** | **L 95** | **U 95** |
| **Adj. for social class** |  |  |  |  |  |  |  |  |  |  |  |  |  |  |  |  |
| Wealth | **1.64** | <0.001 | 1.54 | 1.74 | **1.91** | <0.001 | 1.85 | 1.97 | **1.93** | <0.001 | 1.89 | 1.98 | **1.65** | <0.001 | 1.63 | 1.68 |
|  |  |  |  |  |  |  |  |  |  |  |  |  |  |  |  |  |
| *Social class* |  |  |  |  |  |  |  |  |  |  |  |  |  |  |  |  |
| Intermediate non-manual | **1.01** | 0.894 | 0.92 | 1.09 | **0.98** | 0.335 | 0.94 | 1.02 | **1.00** | 0.94 | 0.95 | 1.04 | **1.01** | 0.902 | 0.89 | 1.14 |
| Lower non-manual | **1.26** | <0.001 | 1.16 | 1.37 | **1.22** | <0.001 | 1.18 | 1.27 | **1.16** | <0.001 | 1.12 | 1.21 | **1.04** | 0.544 | 0.93 | 1.16 |
| Skilled manual | **1.24** | <0.001 | 1.14 | 1.36 | **1.14** | <0.001 | 1.09 | 1.19 | **1.09** | 0.001 | 1.04 | 1.15 | **1.10** | 0.244 | 0.93 | 1.30 |
| Unskilled manual | **1.55** | <0.001 | 1.43 | 1.67 | **1.33** | <0.001 | 1.29 | 1.38 | **1.17** | <0.001 | 1.13 | 1.22 | **1.05** | 0.391 | 0.94 | 1.17 |
| Farmers and farm labourers | **0.96** | 0.776 | 0.71 | 1.29 | **0.96** | 0.487 | 0.87 | 1.07 | **1.06** | 0.143 | 0.98 | 1.14 | **1.33** | <0.001 | 1.17 | 1.51 |
| Self-employed | **1.14** | 0.065 | 0.99 | 1.31 | **1.16** | <0.001 | 1.10 | 1.23 | **1.11** | 0.001 | 1.04 | 1.18 | **1.29** | <0.001 | 1.15 | 1.45 |
| Unclassified employees | **1.77** | <0.001 | 1.61 | 1.95 | **1.72** | <0.001 | 1.63 | 1.81 | **1.58** | <0.001 | 1.51 | 1.66 | **1.30** | <0.001 | 1.18 | 1.43 |
| Economically inactive | **2.86** | <0.001 | 2.64 | 3.10 | **2.76** | <0.001 | 2.66 | 2.86 | **1.94** | <0.001 | 1.87 | 2.01 | **1.62** | <0.001 | 1.47 | 1.78 |

RII=relative index of inequality; P=p value; L 95=lower 95% confidence interval; U95=upper 95% confidence interval

| **WOMEN** | **25-39 years** | | | | **40-54 years** | | | | **55-64 years** | | | | **65-74 years** | | | |
| --- | --- | --- | --- | --- | --- | --- | --- | --- | --- | --- | --- | --- | --- | --- | --- | --- |
|  | **IRR** | **P** | **L 95** | **U 95** | **IRR** | **P** | **L 95** | **U 95** | **IRR** | **P** | **L 95** | **U 95** | **IRR** | **P** | **L 95** | **U 95** |
| **Adj. for all SEP measures** |  |  |  |  |  |  |  |  |  |  |  |  |  |  |  |  |
| Wealth | **1.37** | <0.001 | 1.28 | 1.46 | **1.69** | <0.001 | 1.64 | 1.75 | **1.83** | <0.001 | 1.79 | 1.88 | **1.55** | <0.001 | 1.53 | 1.58 |
|  |  |  |  |  |  |  |  |  |  |  |  |  |  |  |  |  |
| *Individual income* |  |  |  |  |  |  |  |  |  |  |  |  |  |  |  |  |
| 2 | **0.94** | 0.013 | 0.89 | 0.99 | **0.94** | <0.001 | 0.91 | 0.97 | **0.97** | 0.008 | 0.94 | 0.99 | **0.98** | 0.008 | 0.97 | 1.00 |
| 3 | **0.86** | <0.001 | 0.81 | 0.91 | **0.96** | 0.006 | 0.93 | 0.99 | **0.96** | <0.001 | 0.93 | 0.98 | **0.98** | 0.033 | 0.97 | 1.00 |
| 4 | **0.91** | 0.001 | 0.86 | 0.96 | **1.00** | 0.989 | 0.97 | 1.03 | **0.94** | <0.001 | 0.92 | 0.97 | **0.98** | 0.007 | 0.97 | 0.99 |
| Lowest quintile | **0.80** | <0.001 | 0.76 | 0.85 | **0.92** | <0.001 | 0.89 | 0.95 | **0.79** | <0.001 | 0.77 | 0.81 | **0.93** | <0.001 | 0.92 | 0.95 |
|  |  |  |  |  |  |  |  |  |  |  |  |  |  |  |  |  |
| *Household income* |  |  |  |  |  |  |  |  |  |  |  |  |  |  |  |  |
| 2 | **0.99** | 0.73 | 0.94 | 1.05 | **1.06** | <0.001 | 1.02 | 1.09 | **1.07** | <0.001 | 1.04 | 1.09 | **1.05** | <0.001 | 1.03 | 1.06 |
| 3 | **1.09** | 0.003 | 1.03 | 1.15 | **1.16** | <0.001 | 1.13 | 1.20 | **1.14** | <0.001 | 1.11 | 1.17 | **1.12** | <0.001 | 1.11 | 1.14 |
| 4 | **1.31** | <0.001 | 1.24 | 1.38 | **1.29** | <0.001 | 1.25 | 1.32 | **1.26** | <0.001 | 1.23 | 1.29 | **1.21** | <0.001 | 1.19 | 1.22 |
| Lowest quintile | **1.98** | <0.001 | 1.88 | 2.10 | **1.71** | <0.001 | 1.66 | 1.76 | **1.45** | <0.001 | 1.41 | 1.48 | **1.31** | <0.001 | 1.30 | 1.33 |
|  |  |  |  |  |  |  |  |  |  |  |  |  |  |  |  |  |
| *Education* |  |  |  |  |  |  |  |  |  |  |  |  |  |  |  |  |
| Upper secondary | **1.36** | <0.001 | 1.29 | 1.43 | **1.21** | <0.001 | 1.18 | 1.25 | **1.10** | <0.001 | 1.07 | 1.13 | **1.12** | <0.001 | 1.10 | 1.15 |
| Compulsory only | **1.89** | <0.001 | 1.78 | 2.00 | **1.35** | <0.001 | 1.31 | 1.39 | **1.21** | <0.001 | 1.18 | 1.25 | **1.25** | <0.001 | 1.22 | 1.27 |
|  |  |  |  |  |  |  |  |  |  |  |  |  |  |  |  |  |
| *Social class* |  |  |  |  |  |  |  |  |  |  |  |  |  |  |  |  |
| Intermediate non-manual | **1.00** | 0.941 | 0.92 | 1.09 | **0.94** | 0.006 | 0.91 | 0.98 | **0.97** | 0.137 | 0.92 | 1.01 | **0.96** | 0.529 | 0.85 | 1.09 |
| Lower non-manual | **0.97** | 0.49 | 0.89 | 1.06 | **0.99** | 0.802 | 0.95 | 1.04 | **1.03** | 0.257 | 0.98 | 1.08 | **0.92** | 0.149 | 0.82 | 1.03 |
| Skilled manual | **0.95** | 0.305 | 0.86 | 1.05 | **0.92** | 0.001 | 0.87 | 0.97 | **0.96** | 0.128 | 0.90 | 1.01 | **0.96** | 0.61 | 0.81 | 1.13 |
| Unskilled manual | **1.10** | 0.033 | 1.01 | 1.20 | **1.03** | 0.146 | 0.99 | 1.08 | **1.00** | 0.908 | 0.96 | 1.05 | **0.89** | 0.032 | 0.79 | 0.99 |
| Farmers and farm labourers | **0.64** | 0.004 | 0.47 | 0.87 | **0.71** | <0.001 | 0.64 | 0.79 | **0.96** | 0.339 | 0.89 | 1.04 | **1.12** | 0.099 | 0.98 | 1.27 |
| Self-employed | **0.84** | 0.019 | 0.73 | 0.97 | **0.93** | 0.021 | 0.87 | 0.99 | **1.01** | 0.879 | 0.94 | 1.07 | **1.12** | 0.057 | 1.00 | 1.25 |
| Unclassified employees | **1.31** | <0.001 | 1.19 | 1.45 | **1.36** | <0.001 | 1.29 | 1.44 | **1.39** | <0.001 | 1.32 | 1.47 | **1.11** | 0.042 | 1.00 | 1.22 |
| Economically inactive | **2.00** | <0.001 | 1.83 | 2.19 | **2.06** | <0.001 | 1.97 | 2.15 | **1.69** | <0.001 | 1.62 | 1.77 | **1.34** | <0.001 | 1.22 | 1.48 |

RII=relative index of inequality; P=p value; L 95=lower 95% confidence interval; U95=upper 95% confidence interval

**8. Relative Indices of Inequality (RII) by wealth in crude and fully adjusted models, stratified by age group and sex**

| **MEN** | **25-39 years** | | | | **40-54 years** | | | | **55-64 years** | | | | **65-74 years** | | | |
| --- | --- | --- | --- | --- | --- | --- | --- | --- | --- | --- | --- | --- | --- | --- | --- | --- |
|  | **IRR** | **P** | **L 95** | **U 95** | **IRR** | **P** | **L 95** | **U 95** | **IRR** | **P** | **L 95** | **U 95** | **IRR** | **P** | **L 95** | **U 95** |
| Wealth RII (crude) | **2.32** | <0.001 | 2.20 | 2.44 | **2.95** | <0.001 | 2.88 | 3.02 | **2.50** | <0.001 | 2.45 | 2.55 | **1.82** | <0.001 | 1.80 | 1.84 |
|  |  |  |  |  |  |  |  |  |  |  |  |  |  |  |  |  |
| Wealth RII (fully adjusted) | **2.00** | <0.001 | 1.90 | 2.11 | **2.14** | <0.001 | 2.09 | 2.20 | **1.93** | <0.001 | 1.89 | 1.97 | **1.55** | <0.001 | 1.53 | 1.58 |
|  |  |  |  |  |  |  |  |  |  |  |  |  |  |  |  |  |
| *Individual income* |  |  |  |  |  |  |  |  |  |  |  |  |  |  |  |  |
| 2 | **0.99** | 0.719 | 0.94 | 1.04 | **1.08** | <0.001 | 1.05 | 1.11 | **1.07** | <0.001 | 1.04 | 1.09 | **1.05** | <0.001 | 1.03 | 1.06 |
| 3 | **1.10** | <0.001 | 1.05 | 1.16 | **1.10** | <0.001 | 1.07 | 1.14 | **1.08** | <0.001 | 1.06 | 1.11 | **1.02** | 0.005 | 1.01 | 1.04 |
| 4 | **1.25** | <0.001 | 1.19 | 1.32 | **1.20** | <0.001 | 1.17 | 1.24 | **1.14** | <0.001 | 1.11 | 1.16 | **1.05** | <0.001 | 1.04 | 1.07 |
| Lowest quintile | **1.53** | <0.001 | 1.44 | 1.63 | **1.53** | <0.001 | 1.48 | 1.57 | **1.18** | <0.001 | 1.15 | 1.21 | **1.13** | <0.001 | 1.11 | 1.15 |
|  |  |  |  |  |  |  |  |  |  |  |  |  |  |  |  |  |
| *Household income* |  |  |  |  |  |  |  |  |  |  |  |  |  |  |  |  |
| 2 | **0.96** | 0.086 | 0.91 | 1.01 | **1.02** | 0.251 | 0.99 | 1.04 | **1.05** | <0.001 | 1.03 | 1.07 | **1.05** | <0.001 | 1.03 | 1.06 |
| 3 | **1.02** | 0.46 | 0.97 | 1.07 | **1.04** | 0.006 | 1.01 | 1.07 | **1.10** | <0.001 | 1.07 | 1.12 | **1.10** | <0.001 | 1.08 | 1.12 |
| 4 | **1.07** | 0.03 | 1.01 | 1.13 | **1.15** | <0.001 | 1.11 | 1.18 | **1.18** | <0.001 | 1.15 | 1.21 | **1.16** | <0.001 | 1.14 | 1.17 |
| Lowest quintile | **1.27** | <0.001 | 1.19 | 1.36 | **1.49** | <0.001 | 1.44 | 1.54 | **1.54** | <0.001 | 1.50 | 1.58 | **1.40** | <0.001 | 1.38 | 1.42 |
|  |  |  |  |  |  |  |  |  |  |  |  |  |  |  |  |  |
| *Education* |  |  |  |  |  |  |  |  |  |  |  |  |  |  |  |  |
| Upper secondary | **1.56** | <0.001 | 1.50 | 1.63 | **1.21** | <0.001 | 1.18 | 1.24 | **1.06** | <0.001 | 1.04 | 1.09 | **1.07** | <0.001 | 1.05 | 1.09 |
| Compulsory only | **2.01** | <0.001 | 1.92 | 2.10 | **1.30** | <0.001 | 1.27 | 1.33 | **1.13** | <0.001 | 1.11 | 1.15 | **1.14** | <0.001 | 1.12 | 1.15 |

RII=relative index of inequality; P=p value; L 95=lower 95% confidence interval; U95=upper 95% confidence interval

Crude models are adjusted for age and follow-up period. Fully adjusted models are additionally adjusted for region (not shown), four measures of socioeconomic position, number of children within household, number of other adults within household and country of birth.

| **MEN** | **25-39 years** | | | | **40-54 years** | | | | **55-64 years** | | | | **65-74 years** | | | |
| --- | --- | --- | --- | --- | --- | --- | --- | --- | --- | --- | --- | --- | --- | --- | --- | --- |
|  | **IRR** | **P** | **L 95** | **U 95** | **IRR** | **P** | **L 95** | **U 95** | **IRR** | **P** | **L 95** | **U 95** | **IRR** | **P** | **L 95** | **U 95** |
| *Social class* |  |  |  |  |  |  |  |  |  |  |  |  |  |  |  |  |
| Intermediate non-manual | **1.07** | 0.057 | 1.00 | 1.14 | **0.99** | 0.692 | 0.97 | 1.02 | **0.99** | 0.545 | 0.97 | 1.02 | **0.99** | 0.739 | 0.93 | 1.05 |
| Lower non-manual | **0.99** | 0.825 | 0.92 | 1.07 | **1.06** | <0.001 | 1.03 | 1.10 | **1.03** | 0.066 | 1.00 | 1.06 | **1.04** | 0.277 | 0.97 | 1.10 |
| Skilled manual | **1.10** | 0.004 | 1.03 | 1.17 | **1.07** | <0.001 | 1.04 | 1.11 | **1.02** | 0.223 | 0.99 | 1.05 | **1.00** | 0.996 | 0.93 | 1.07 |
| Unskilled manual | **1.16** | <0.001 | 1.09 | 1.24 | **1.11** | <0.001 | 1.08 | 1.14 | **1.06** | <0.001 | 1.03 | 1.09 | **1.05** | 0.073 | 1.00 | 1.12 |
| Farmers and farm labourers | **0.76** | <0.001 | 0.66 | 0.87 | **0.70** | <0.001 | 0.66 | 0.75 | **0.82** | <0.001 | 0.78 | 0.85 | **1.01** | 0.657 | 0.96 | 1.06 |
| Self-employed | **0.87** | 0.002 | 0.80 | 0.95 | **0.89** | <0.001 | 0.86 | 0.93 | **0.95** | 0.004 | 0.92 | 0.98 | **1.11** | <0.001 | 1.06 | 1.16 |
| Unclassified employees | **1.41** | <0.001 | 1.32 | 1.51 | **1.32** | <0.001 | 1.27 | 1.36 | **1.30** | <0.001 | 1.25 | 1.34 | **1.12** | <0.001 | 1.08 | 1.17 |
| Economically inactive | **2.23** | <0.001 | 2.09 | 2.39 | **1.88** | <0.001 | 1.83 | 1.95 | **1.54** | <0.001 | 1.50 | 1.58 | **1.30** | <0.001 | 1.25 | 1.35 |
|  |  |  |  |  |  |  |  |  |  |  |  |  |  |  |  |  |
| *No. of kids in household* |  |  |  |  |  |  |  |  |  |  |  |  |  |  |  |  |
| 1 child | **0.70** | <0.001 | 0.67 | 0.74 | **0.75** | <0.001 | 0.74 | 0.77 | **0.85** | <0.001 | 0.83 | 0.88 | **0.92** | 0.005 | 0.87 | 0.97 |
| 2-3 children | **0.59** | <0.001 | 0.57 | 0.62 | **0.63** | <0.001 | 0.61 | 0.64 | **0.80** | <0.001 | 0.76 | 0.84 | **0.81** | 0.001 | 0.72 | 0.91 |
| 4+ children | **0.64** | <0.001 | 0.58 | 0.70 | **0.64** | <0.001 | 0.59 | 0.69 | **0.83** | 0.065 | 0.67 | 1.01 | **0.92** | 0.714 | 0.59 | 1.44 |
|  |  |  |  |  |  |  |  |  |  |  |  |  |  |  |  |  |
| *No. of other adults in household* |  |  |  |  |  |  |  |  |  |  |  |  |  |  |  |  |
| 1 adult | **1.31** | <0.001 | 1.24 | 1.38 | **0.96** | <0.001 | 0.95 | 0.98 | **0.99** | 0.524 | 0.98 | 1.01 | **1.07** | <0.001 | 1.05 | 1.09 |
| 2+ adults | **1.20** | <0.001 | 1.09 | 1.31 | **0.92** | <0.001 | 0.89 | 0.95 | **0.95** | 0.009 | 0.92 | 0.99 | **1.03** | 0.218 | 0.98 | 1.07 |
|  |  |  |  |  |  |  |  |  |  |  |  |  |  |  |  |  |
| *Country of birth* |  |  |  |  |  |  |  |  |  |  |  |  |  |  |  |  |
| Born abroad | **0.88** | <0.001 | 0.85 | 0.92 | **0.94** | <0.001 | 0.92 | 0.96 | **0.97** | 0.002 | 0.95 | 0.99 | **1.00** | 0.93 | 0.98 | 1.02 |

RII=relative index of inequality; P=p value; L 95=lower 95% confidence interval; U95=upper 95% confidence interval

| **WOMEN** | **25-39 years** | | | | **40-54 years** | | | | **55-64 years** | | | | **65-74 years** | | | |
| --- | --- | --- | --- | --- | --- | --- | --- | --- | --- | --- | --- | --- | --- | --- | --- | --- |
|  | **IRR** | **P** | **L 95** | **U 95** | **IRR** | **P** | **L 95** | **U 95** | **IRR** | **P** | **L 95** | **U 95** | **IRR** | **P** | **L 95** | **U 95** |
| Wealth RII (crude) | **1.84** | <0.001 | 1.73 | 1.95 | **2.31** | <0.001 | 2.24 | 2.38 | **2.24** | <0.001 | 2.19 | 2.29 | **1.69** | <0.001 | 1.67 | 1.71 |
|  |  |  |  |  |  |  |  |  |  |  |  |  |  |  |  |  |
| Wealth RII (adjusted) | **1.39** | <0.001 | 1.30 | 1.48 | **1.74** | <0.001 | 1.69 | 1.80 | **1.82** | <0.001 | 1.78 | 1.87 | **1.53** | <0.001 | 1.50 | 1.55 |
|  |  |  |  |  |  |  |  |  |  |  |  |  |  |  |  |  |
| *Individual income* |  |  |  |  |  |  |  |  |  |  |  |  |  |  |  |  |
| 2 | **0.88** | <0.001 | 0.84 | 0.93 | **0.93** | <0.001 | 0.91 | 0.96 | **0.97** | 0.034 | 0.95 | 1.00 | **0.98** | 0.019 | 0.97 | 1.00 |
| 3 | **0.83** | <0.001 | 0.78 | 0.88 | **0.94** | <0.001 | 0.91 | 0.97 | **0.97** | 0.008 | 0.94 | 0.99 | **0.98** | 0.01 | 0.96 | 1.00 |
| 4 | **0.87** | <0.001 | 0.82 | 0.92 | **0.98** | 0.188 | 0.95 | 1.01 | **0.96** | <0.001 | 0.93 | 0.98 | **0.98** | 0.005 | 0.96 | 0.99 |
| Lowest quintile | **0.79** | <0.001 | 0.75 | 0.84 | **0.91** | <0.001 | 0.88 | 0.94 | **0.80** | <0.001 | 0.78 | 0.82 | **0.94** | <0.001 | 0.92 | 0.95 |
|  |  |  |  |  |  |  |  |  |  |  |  |  |  |  |  |  |
| *Household income* |  |  |  |  |  |  |  |  |  |  |  |  |  |  |  |  |
| 2 | **1.03** | 0.367 | 0.97 | 1.09 | **1.04** | 0.008 | 1.01 | 1.08 | **1.09** | <0.001 | 1.07 | 1.12 | **1.09** | <0.001 | 1.08 | 1.11 |
| 3 | **1.08** | 0.01 | 1.02 | 1.14 | **1.11** | <0.001 | 1.08 | 1.15 | **1.18** | <0.001 | 1.15 | 1.21 | **1.18** | <0.001 | 1.16 | 1.20 |
| 4 | **1.10** | 0.001 | 1.04 | 1.17 | **1.19** | <0.001 | 1.16 | 1.23 | **1.30** | <0.001 | 1.27 | 1.34 | **1.27** | <0.001 | 1.25 | 1.29 |
| Lowest quintile | **1.36** | <0.001 | 1.27 | 1.46 | **1.47** | <0.001 | 1.42 | 1.52 | **1.50** | <0.001 | 1.46 | 1.54 | **1.39** | <0.001 | 1.36 | 1.41 |
|  |  |  |  |  |  |  |  |  |  |  |  |  |  |  |  |  |
| *Education* |  |  |  |  |  |  |  |  |  |  |  |  |  |  |  |  |
| Upper secondary | **1.39** | <0.001 | 1.32 | 1.46 | **1.18** | <0.001 | 1.15 | 1.22 | **1.10** | <0.001 | 1.07 | 1.13 | **1.12** | <0.001 | 1.09 | 1.14 |
| Compulsory only | **1.94** | <0.001 | 1.84 | 2.06 | **1.30** | <0.001 | 1.26 | 1.34 | **1.22** | <0.001 | 1.18 | 1.25 | **1.24** | <0.001 | 1.21 | 1.26 |

RII=relative index of inequality; P=p value; L 95=lower 95% confidence interval; U95=upper 95% confidence interval

Crude models are adjusted for age and follow-up period. Fully adjusted models are additionally adjusted for region (not shown), four measures of socioeconomic position, number of children within household, number of other adults within household and country of birth.

| **WOMEN** | **25-39 years** | |  |  | **40-54 years** | |  |  | **55-64 years** | |  |  | **65-74 years** | |  |  |
| --- | --- | --- | --- | --- | --- | --- | --- | --- | --- | --- | --- | --- | --- | --- | --- | --- |
|  | **IRR** | **P** | **L 95** | **U 95** | **IRR** | **P** | **L 95** | **U 95** | **IRR** | **P** | **L 95** | **U 95** | **IRR** | **P** | **L 95** | **U 95** |
| *Social class* |  |  |  |  |  |  |  |  |  |  |  |  |  |  |  |  |
| Intermediate non-manual | **1.07** | 0.104 | 0.99 | 1.17 | **0.97** | 0.086 | 0.93 | 1.01 | **0.97** | 0.186 | 0.93 | 1.01 | **0.96** | 0.47 | 0.84 | 1.08 |
| Lower non-manual | **1.03** | 0.489 | 0.94 | 1.13 | **1.01** | 0.707 | 0.97 | 1.05 | **1.02** | 0.431 | 0.97 | 1.07 | **0.92** | 0.151 | 0.82 | 1.03 |
| Skilled manual | **1.06** | 0.246 | 0.96 | 1.17 | **0.96** | 0.134 | 0.91 | 1.01 | **0.97** | 0.259 | 0.92 | 1.02 | **0.95** | 0.571 | 0.81 | 1.13 |
| Unskilled manual | **1.25** | <0.001 | 1.14 | 1.37 | **1.10** | <0.001 | 1.05 | 1.14 | **1.02** | 0.523 | 0.97 | 1.06 | **0.88** | 0.023 | 0.79 | 0.98 |
| Farmers and farm labourers | **0.88** | 0.406 | 0.65 | 1.19 | **0.83** | 0.001 | 0.74 | 0.92 | **0.98** | 0.538 | 0.90 | 1.06 | **1.10** | 0.15 | 0.97 | 1.25 |
| Self-employed | **0.97** | 0.689 | 0.84 | 1.12 | **0.98** | 0.579 | 0.92 | 1.05 | **1.01** | 0.7 | 0.95 | 1.08 | **1.12** | 0.054 | 1.00 | 1.25 |
| Unclassified employees | **1.48** | <0.001 | 1.34 | 1.63 | **1.43** | <0.001 | 1.36 | 1.51 | **1.40** | <0.001 | 1.33 | 1.48 | **1.10** | 0.071 | 0.99 | 1.21 |
| Economically inactive | **2.38** | <0.001 | 2.17 | 2.61 | **2.26** | <0.001 | 2.16 | 2.37 | **1.71** | <0.001 | 1.64 | 1.79 | **1.32** | <0.001 | 1.20 | 1.46 |
|  |  |  |  |  |  |  |  |  |  |  |  |  |  |  |  |  |
| *No. of kids in household* |  |  |  |  |  |  |  |  |  |  |  |  |  |  |  |  |
| 1 child | **0.72** | <0.001 | 0.68 | 0.75 | **0.78** | <0.001 | 0.77 | 0.80 | **0.87** | <0.001 | 0.82 | 0.93 | **0.74** | 0.071 | 0.53 | 1.03 |
| 2-3 children | **0.53** | <0.001 | 0.50 | 0.56 | **0.63** | <0.001 | 0.61 | 0.65 | **0.79** | 0.039 | 0.63 | 0.99 | **1.18** | 0.745 | 0.44 | 3.12 |
| 4+ children | **0.48** | <0.001 | 0.44 | 0.54 | **0.45** | <0.001 | 0.39 | 0.51 | **0.00** | <0.001 | 0.00 | 0.00 |  |  |  |  |
|  |  |  |  |  |  |  |  |  |  |  |  |  |  |  |  |  |
| *No. of other adults in household* |  |  |  |  |  |  |  |  |  |  |  |  |  |  |  |  |
| 1 adult | **1.17** | <0.001 | 1.09 | 1.24 | **0.93** | <0.001 | 0.91 | 0.95 | **1.07** | <0.001 | 1.05 | 1.10 | **1.18** | <0.001 | 1.16 | 1.20 |
| 2+ adults | **1.13** | 0.134 | 0.96 | 1.33 | **0.88** | <0.001 | 0.85 | 0.92 | **1.06** | 0.024 | 1.01 | 1.11 | **1.26** | <0.001 | 1.19 | 1.34 |
|  |  |  |  |  |  |  |  |  |  |  |  |  |  |  |  |  |
| *Country of birth* |  |  |  |  |  |  |  |  |  |  |  |  |  |  |  |  |
| Born abroad | **0.85** | <0.001 | 0.81 | 0.89 | **0.82** | <0.001 | 0.80 | 0.84 | **0.91** | <0.001 | 0.89 | 0.93 | **1.00** | 0.693 | 0.99 | 1.02 |

RII=relative index of inequality; P=p value; L 95=lower 95% confidence interval; U95=upper 95% confidence interval

**9. Inequalities in all-cause mortality by wealth tax paid (assessed by a categorical variable), adjusted for age group and follow-up period**

|  |  | **MEN** | | | | **WOMEN** | | | |
| --- | --- | --- | --- | --- | --- | --- | --- | --- | --- |
|  |  | **IRR** | **P** | **L95** | **U95** | **IRR** | **P** | **L95** | **U95** |
| **25-39 years** | **Wealth tax paid** | **1** |  |  |  | **1** |  |  |  |
|  | **None paid** | **1.47** | <0.001 | 1.43 | 1.51 | **1.31** | <0.001 | 1.27 | 1.36 |
| **40-54 years** | **Wealth tax paid** | **1** |  |  |  | **1** |  |  |  |
|  | **None paid** | **1.65** | <0.001 | 1.63 | 1.67 | **1.54** | <0.001 | 1.51 | 1.56 |

|  |  | **MEN** | | | | **WOMEN** | | | |
| --- | --- | --- | --- | --- | --- | --- | --- | --- | --- |
|  |  | **IRR** | **P** | **L95** | **U95** | **IRR** | **P** | **L95** | **U95** |
| **55-64 years** | **Quartile paying most wealth tax** | **1** |  |  |  | **1** |  |  |  |
|  | **2** | **1.21** | <0.001 | 1.19 | 1.22 | **1.16** | <0.001 | 1.13 | 1.18 |
|  | **3** | **1.63** | <0.001 | 1.60 | 1.65 | **1.39** | <0.001 | 1.37 | 1.42 |
|  | **Least** | **1.97** | <0.001 | 1.94 | 2.00 | **1.85** | <0.001 | 1.82 | 1.89 |
| **65-74 years** | **Quartile paying most wealth tax** | **1** |  |  |  | **1** |  |  |  |
|  | **2** | **1.13** | <0.001 | 1.12 | 1.15 | **1.14** | <0.001 | 1.13 | 1.15 |
|  | **3** | **1.30** | <0.001 | 1.29 | 1.32 | **1.26** | <0.001 | 1.24 | 1.27 |
|  | **Least** | **1.66** | <0.001 | 1.64 | 1.68 | **1.53** | <0.001 | 1.51 | 1.55 |
| **75-84 years** | **Quartile paying most wealth tax** | **1** |  |  |  | **1** |  |  |  |
|  | **2** | **1.10** | <0.001 | 1.09 | 1.11 | **1.08** | <0.001 | 1.07 | 1.09 |
|  | **3** | **1.21** | <0.001 | 1.19 | 1.22 | **1.11** | <0.001 | 1.10 | 1.13 |
|  | **Least** | **1.45** | <0.001 | 1.43 | 1.46 | **1.30** | <0.001 | 1.29 | 1.31 |
| **85+ years** | **Quartile paying most wealth tax** | **1** |  |  |  | **1** |  |  |  |
|  | **2** | **1.08** | <0.001 | 1.05 | 1.10 | **1.02** | 0.032 | 1.00 | 1.03 |
|  | **3** | **1.15** | <0.001 | 1.12 | 1.17 | **1.04** | <0.001 | 1.02 | 1.06 |
|  | **Least** | **1.49** | <0.001 | 1.45 | 1.52 | **1.36** | <0.001 | 1.34 | 1.38 |

IRR=Incidence rate ratio; P=p value; L 95=lower 95% confidence interval; U95=upper 95% confidence interval

**10. Inequalities in all-cause mortality by wealth tax paid (assessed by a categorical variable), adjusted for a) age group and follow-up period, b) four measures of socioeconomic position, and c) all covariates**

|  |  | **MEN** | | | | **WOMEN** | | | |
| --- | --- | --- | --- | --- | --- | --- | --- | --- | --- |
|  |  | **IRR** | **P** | **L95** | **U95** | **IRR** | **P** | **L95** | **U95** |
| **25-39 years** | | | | | | | | | |
| **Crude** | Wealth tax paid | **1** |  |  |  | **1** |  |  |  |
|  | None paid | **1.49** | <0.001 | 1.45 | 1.53 | **1.33** | <0.001 | 1.29 | 1.37 |
| **Adj. for all SEP measures** | Wealth tax paid | **1** |  |  |  | **1** |  |  |  |
|  | None paid | **1.33** | <0.001 | 1.30 | 1.37 | **1.17** | <0.001 | 1.13 | 1.21 |
| **Fully adjusted** | Wealth tax paid | **1** |  |  |  | **1** |  |  |  |
|  | None paid | **1.41** | <0.001 | 1.37 | 1.44 | **1.18** | <0.001 | 1.14 | 1.22 |
| **40-54 years** | | | | | | | | | |
| **Crude** | Wealth tax paid | **1** |  |  |  | **1** |  |  |  |
|  | None paid | **1.65** | <0.001 | 1.63 | 1.67 | **1.54** | <0.001 | 1.52 | 1.57 |
| **Adj. for all SEP measures** | Wealth tax paid | **1** |  |  |  | **1** |  |  |  |
|  | None paid | **1.42** | <0.001 | 1.40 | 1.44 | **1.33** | <0.001 | 1.31 | 1.35 |
| **Fully adjusted** | Wealth tax paid | **1** |  |  |  | **1** |  |  |  |
|  | None paid | **1.43** | <0.001 | 1.42 | 1.45 | **1.35** | <0.001 | 1.32 | 1.37 |

IRR=Incidence rate ratio; P=p value; L 95=lower 95% confidence interval; U95=upper 95% confidence interval

Crude models are adjusted for age and follow-up period. Models are then adjusted for four measures of socioeconomic position (education, individual income, household income, social class). Fully adjusted models are adjusted for region, four measures of socioeconomic position, number of children within household, number of other adults within household and country of birth.

|  |  | **MEN** | | | | **WOMEN** | | | |
| --- | --- | --- | --- | --- | --- | --- | --- | --- | --- |
|  |  | **IRR** | **P** | **L95** | **U95** | **IRR** | **P** | **L95** | **U95** |
| **55-64 years** | | | | | | | | | |
| **Crude** | Quartile paying most wealth tax | **1** |  |  |  | **1** |  |  |  |
|  | 2 | **1.21** | <0.001 | 1.19 | 1.23 | **1.16** | <0.001 | 1.14 | 1.18 |
|  | 3 | **1.61** | <0.001 | 1.58 | 1.63 | **1.39** | <0.001 | 1.36 | 1.42 |
|  | Least | **1.93** | <0.001 | 1.90 | 1.96 | **1.82** | <0.001 | 1.79 | 1.86 |
| **Adj. for all SEP measures** | Quartile paying most wealth tax | **1** |  |  |  | **1** |  |  |  |
|  | 2 | **1.11** | <0.001 | 1.09 | 1.13 | **1.11** | <0.001 | 1.08 | 1.13 |
|  | 3 | **1.34** | <0.001 | 1.32 | 1.36 | **1.26** | <0.001 | 1.24 | 1.29 |
|  | Least | **1.60** | <0.001 | 1.57 | 1.62 | **1.56** | <0.001 | 1.54 | 1.59 |
| **Fully adjusted** | Quartile paying most wealth tax | **1** |  |  |  | **1** |  |  |  |
|  | 2 | **1.12** | <0.001 | 1.10 | 1.14 | **1.11** | <0.001 | 1.09 | 1.13 |
|  | 3 | **1.35** | <0.001 | 1.33 | 1.37 | **1.27** | <0.001 | 1.24 | 1.29 |
|  | Least | **1.60** | <0.001 | 1.58 | 1.63 | **1.56** | <0.001 | 1.53 | 1.59 |

IRR=Incidence rate ratio; P=p value; L 95=lower 95% confidence interval; U95=upper 95% confidence interval

Crude models are adjusted for age and follow-up period. Models are then adjusted for four measures of socioeconomic position (education, individual income, household income, social class). Fully adjusted models are adjusted for region, four measures of socioeconomic position, number of children within household, number of other adults within household and country of birth.

|  |  | **MEN** | | | | **WOMEN** | | | |
| --- | --- | --- | --- | --- | --- | --- | --- | --- | --- |
|  |  | **IRR** | **P** | **L95** | **U95** | **IRR** | **P** | **L95** | **U95** |
| **65-74 years** | | | | | | | | | |
| **Crude** | Quartile paying most wealth tax | **1** |  |  |  | **1** |  |  |  |
|  | 2 | **1.13** | <0.001 | 1.12 | 1.14 | **1.14** | <0.001 | 1.13 | 1.15 |
|  | 3 | **1.29** | <0.001 | 1.28 | 1.30 | **1.26** | <0.001 | 1.24 | 1.27 |
|  | Least | **1.58** | <0.001 | 1.56 | 1.60 | **1.49** | <0.001 | 1.47 | 1.50 |
| **Adj. for all SEP measures** | Quartile paying most wealth tax | **1** |  |  |  | **1** |  |  |  |
|  | 2 | **1.05** | <0.001 | 1.04 | 1.06 | **1.09** | <0.001 | 1.07 | 1.10 |
|  | 3 | **1.16** | <0.001 | 1.15 | 1.18 | **1.18** | <0.001 | 1.17 | 1.19 |
|  | Least | **1.40** | <0.001 | 1.38 | 1.41 | **1.39** | <0.001 | 1.37 | 1.40 |
| **Fully adjusted** | Quartile paying most wealth tax | **1** |  |  |  | **1** |  |  |  |
|  | 2 | **1.05** | <0.001 | 1.04 | 1.07 | **1.08** | <0.001 | 1.07 | 1.09 |
|  | 3 | **1.16** | <0.001 | 1.15 | 1.17 | **1.17** | <0.001 | 1.16 | 1.18 |
|  | Least | **1.39** | <0.001 | 1.38 | 1.41 | **1.37** | <0.001 | 1.35 | 1.38 |

IRR=Incidence rate ratio; P=p value; L 95=lower 95% confidence interval; U95=upper 95% confidence interval

Crude models are adjusted for age and follow-up period. Models are then adjusted for four measures of socioeconomic position (education, individual income, household income, social class). Fully adjusted models are adjusted for region, four measures of socioeconomic position, number of children within household, number of other adults within household and country of birth.

**11. Relative and slope indices of inequality for cause-specific mortality by wealth, individual income and household income (adjusted for age and follow-up period)**

| **MEN** | **Wealth** | | | | | **Individual income** | | | | | **Household income** | | | | |
| --- | --- | --- | --- | --- | --- | --- | --- | --- | --- | --- | --- | --- | --- | --- | --- |
|  | **RII** | **P** | **L 95%** | **U 95%** | **SII** | **RII** | **P** | **L 95%** | **U 95%** | **SII** | **RII** | **P** | **L 95%** | **U 95%** | **SII** |
| Infection | **2.23** | <0.001 | 2.07 | 2.41 | 26.84 | **3.05** | <0.001 | 2.76 | 3.35 | 35.57 | **3.72** | <0.001 | 3.35 | 4.14 | 40.57 |
| Cancer | **1.78** | <0.001 | 1.75 | 1.81 | 400.32 | **1.41** | <0.001 | 1.39 | 1.44 | 244.98 | **1.53** | <0.001 | 1.50 | 1.56 | 298.43 |
| Lung cancer | **3.64** | <0.001 | 3.51 | 3.78 | 116.38 | **1.91** | <0.001 | 1.82 | 1.99 | 63.72 | **2.22** | <0.001 | 2.12 | 2.33 | 77.51 |
| Stomach cancer | **2.20** | <0.001 | 2.08 | 2.33 | 39.28 | **2.09** | <0.001 | 1.96 | 2.23 | 36.93 | **2.11** | <0.001 | 1.96 | 2.27 | 37.34 |
| Colon cancer | **1.50** | <0.001 | 1.43 | 1.57 | 33.84 | **1.34** | <0.001 | 1.27 | 1.42 | 24.67 | **1.46** | <0.001 | 1.38 | 1.55 | 31.90 |
| Other cancers | **1.63** | <0.001 | 1.59 | 1.67 | 139.01 | **1.37** | <0.001 | 1.33 | 1.41 | 90.33 | **1.47** | <0.001 | 1.43 | 1.52 | 111.48 |
| Diabetes | **2.98** | <0.001 | 2.82 | 3.15 | 60.34 | **4.04** | <0.001 | 3.79 | 4.30 | 73.06 | **5.30** | <0.001 | 4.92 | 5.72 | 82.73 |
| Dementia | **1.45** | <0.001 | 1.38 | 1.52 | 38.76 | **1.53** | <0.001 | 1.44 | 1.62 | 44.11 | **1.57** | <0.001 | 1.47 | 1.67 | 46.68 |
| Circulatory diseases | **2.14** | <0.001 | 2.11 | 2.16 | 1314.30 | **2.42** | <0.001 | 2.38 | 2.45 | 1503.97 | **2.92** | <0.001 | 2.87 | 2.96 | 1776.15 |
| IHD | **2.23** | <0.001 | 2.20 | 2.26 | 729.65 | **2.56** | <0.001 | 2.51 | 2.61 | 839.78 | **3.10** | <0.001 | 3.03 | 3.16 | 980.37 |
| Stroke | **1.92** | <0.001 | 1.87 | 1.97 | 218.58 | **2.21** | <0.001 | 2.14 | 2.29 | 261.48 | **2.54** | <0.001 | 2.44 | 2.63 | 300.71 |
| Other circulatory diseases | **2.10** | <0.001 | 2.05 | 2.14 | 361.93 | **2.26** | <0.001 | 2.20 | 2.33 | 395.30 | **2.83** | <0.001 | 2.74 | 2.92 | 487.68 |
| Respiratory disease | **3.02** | <0.001 | 2.93 | 3.10 | 339.65 | **3.23** | <0.001 | 3.11 | 3.36 | 356.97 | **4.20** | <0.001 | 4.02 | 4.39 | 416.35 |
| Alcohol | **23.57** | <0.001 | 20.86 | 26.64 | 22.44 | **13.49** | <0.001 | 12.51 | 14.55 | 21.05 | **61.22** | <0.001 | 54.48 | 68.79 | 23.64 |
| Drugs | **109.52** | <0.001 | 55.29 | 216.94 | 2.19 | **60.61** | <0.001 | 47.27 | 77.71 | 2.16 | **88.68** | <0.001 | 57.02 | 137.91 | 2.18 |
| Accidents and violence | **1.96** | <0.001 | 1.88 | 2.05 | 50.50 | **3.33** | <0.001 | 3.18 | 3.49 | 83.62 | **4.55** | <0.001 | 4.31 | 4.79 | 99.27 |
| Road traffic incidents | **1.57** | <0.001 | 1.35 | 1.83 | 2.32 | **2.29** | <0.001 | 1.94 | 2.70 | 4.10 | **2.29** | <0.001 | 1.93 | 2.70 | 4.09 |
| Suicide | **1.37** | <0.001 | 1.26 | 1.50 | 5.08 | **2.94** | <0.001 | 2.71 | 3.19 | 15.95 | **3.75** | <0.001 | 3.42 | 4.10 | 18.74 |
| Homicide | **9.53** | <0.001 | 6.04 | 15.04 | 1.31 | **14.24** | <0.001 | 10.39 | 19.52 | 1.41 | **18.76** | <0.001 | 12.21 | 28.84 | 1.45 |
| Other accidents and violence | **2.29** | <0.001 | 2.17 | 2.41 | 43.38 | **3.60** | <0.001 | 3.39 | 3.83 | 62.63 | **5.39** | <0.001 | 5.03 | 5.78 | 76.12 |
| Prostate cancer | **1.20** | <0.001 | 1.16 | 1.24 | 32.93 | **1.07** | 0.002 | 1.02 | 1.11 | 11.66 | **1.08** | 0.001 | 1.03 | 1.13 | 14.01 |

| **WOMEN** | **RII** | **P** | **L 95%** | **U 95%** | **SII** | **RII** | **P** | **L 95%** | **U 95%** | **SII** | **RII** | **P** | **L 95%** | **U 95%** | **SII** |
| --- | --- | --- | --- | --- | --- | --- | --- | --- | --- | --- | --- | --- | --- | --- | --- |
| Infection | **1.98** | <0.001 | 1.83 | 2.15 | 18.70 | **1.82** | <0.001 | 1.63 | 2.04 | 16.55 | **2.74** | <0.001 | 2.44 | 3.06 | 26.36 |
| Cancer | **1.61** | <0.001 | 1.58 | 1.64 | 213.91 | **1.11** | <0.001 | 1.08 | 1.13 | 46.38 | **1.47** | <0.001 | 1.44 | 1.50 | 174.75 |
| Lung cancer | **3.47** | <0.001 | 3.29 | 3.65 | 57.15 | **0.91** | 0.001 | 0.86 | 0.96 | -4.82 | **2.16** | <0.001 | 2.05 | 2.29 | 38.09 |
| Stomach cancer | **1.92** | <0.001 | 1.77 | 2.10 | 14.93 | **1.27** | <0.001 | 1.14 | 1.41 | 5.62 | **1.75** | <0.001 | 1.58 | 1.94 | 12.86 |
| Colon cancer | **1.37** | <0.001 | 1.30 | 1.45 | 19.12 | **1.12** | 0.001 | 1.05 | 1.19 | 6.81 | **1.28** | <0.001 | 1.20 | 1.36 | 15.00 |
| Other cancers | **1.52** | <0.001 | 1.47 | 1.56 | 85.37 | **1.18** | <0.001 | 1.14 | 1.22 | 34.49 | **1.39** | <0.001 | 1.34 | 1.43 | 67.72 |
| Diabetes | **4.04** | <0.001 | 3.80 | 4.30 | 58.48 | **3.33** | <0.001 | 3.05 | 3.63 | 52.14 | **4.00** | <0.001 | 3.66 | 4.38 | 58.20 |
| Dementia | **1.13** | <0.001 | 1.09 | 1.17 | 18.46 | **1.41** | <0.001 | 1.34 | 1.49 | 51.43 | **1.55** | <0.001 | 1.48 | 1.63 | 65.36 |
| Circulatory diseases | **1.98** | <0.001 | 1.95 | 2.00 | 871.00 | **1.70** | <0.001 | 1.67 | 1.73 | 685.22 | **2.33** | <0.001 | 2.29 | 2.38 | 1061.47 |
| IHD | **2.27** | <0.001 | 2.22 | 2.31 | 436.78 | **1.82** | <0.001 | 1.77 | 1.87 | 326.79 | **2.60** | <0.001 | 2.52 | 2.67 | 500.46 |
| Stroke | **1.68** | <0.001 | 1.64 | 1.73 | 169.06 | **1.70** | <0.001 | 1.64 | 1.77 | 172.75 | **2.08** | <0.001 | 2.00 | 2.15 | 231.84 |
| Other circulatory diseases | **1.86** | <0.001 | 1.82 | 1.91 | 260.71 | **1.53** | <0.001 | 1.48 | 1.58 | 181.38 | **2.21** | <0.001 | 2.14 | 2.29 | 326.09 |
| Respiratory disease | **2.61** | <0.001 | 2.52 | 2.70 | 185.12 | **1.55** | <0.001 | 1.48 | 1.62 | 89.20 | **3.26** | <0.001 | 3.11 | 3.43 | 220.62 |
| Alcohol | **21.76** | <0.001 | 16.11 | 29.39 | 4.26 | **2.35** | <0.001 | 1.82 | 3.03 | 1.88 | **24.77** | <0.001 | 19.02 | 32.26 | 4.31 |
| Drugs | **41.83** | <0.001 | 14.76 | 118.51 | 0.51 | **4.69** | <0.001 | 2.04 | 10.78 | 0.34 | **31.31** | <0.001 | 13.78 | 71.12 | 0.50 |
| Accidents and violence | **1.69** | <0.001 | 1.59 | 1.79 | 23.67 | **1.17** | <0.001 | 1.09 | 1.26 | 7.35 | **2.74** | <0.001 | 2.54 | 2.95 | 43.04 |
| Road traffic incidents | **1.12** | 0.375 | 0.87 | 1.43 | 0.22 | **0.82** | 0.179 | 0.62 | 1.09 | -0.39 | **1.09** | 0.536 | 0.83 | 1.42 | 0.17 |
| Suicide | **1.83** | <0.001 | 1.58 | 2.12 | 3.44 | **1.16** | 0.066 | 0.99 | 1.35 | 0.86 | **4.09** | <0.001 | 3.52 | 4.75 | 7.14 |
| Homicide | **2.15** | 0.013 | 1.17 | 3.93 | 0.29 | **1.15** | 0.678 | 0.59 | 2.25 | 0.06 | **2.30** | 0.01 | 1.22 | 4.32 | 0.31 |
| Other accidents and violence | **1.71** | <0.001 | 1.60 | 1.83 | 19.92 | **1.22** | <0.001 | 1.12 | 1.33 | 7.56 | **2.64** | <0.001 | 2.41 | 2.89 | 34.28 |
| Breast cancer | **1.24** | <0.001 | 1.18 | 1.30 | 14.18 | **1.03** | 0.319 | 0.97 | 1.09 | 1.93 | **1.27** | <0.001 | 1.20 | 1.34 | 16.15 |
| Female reproductive cancers | **1.32** | <0.001 | 1.25 | 1.40 | 13.15 | **1.15** | <0.001 | 1.08 | 1.23 | 6.79 | **1.48** | <0.001 | 1.38 | 1.57 | 18.22 |
